# P2X7 receptor-mediated IL-1β release by human brain tissue: the impact of CNS-penetrant potential therapeutics

**DOI:** 10.1093/brain/awag068

**Published:** 2026-02-19

**Authors:** Jamie Cowley, Neale Harrison, Ashley Pegg, Hujo Chan, Zhi Li, Arshpreet Kaur, Emma Welsh, Alex Brewin, Juan Carlos Yam-Puc, Grace Fensome, Edward Broome, Ines M Morano, Elizabeth Jinks, Gillian Grafton, Alison J Cooper, Zubair Ahmed, Andrew R Stevens, Freya G Anderson, Victoria Wykes, Ramesh Chelvarajah, Georgios Tsermoulas, Ismail Ughratdar, Athanasios Zisakis, Philip J O’Halloran, David J Davies, Thomas Land, Yasir A Chowdhury, Zenab Sher, Kamal M Yakoub, Colin Bergin, Luke Galloway, Rachel Upthegrove, Catherine A Brady, John Gordon, Omar Qureshi, Antonio Belli, Nicholas M Barnes

**Affiliations:** Neuropharmacology Research Group, School of Health Sciences, College of Medicine and Health, University of Birmingham, Birmingham B15 2TT, UK; Neuropharmacology Research Group, School of Health Sciences, College of Medicine and Health, University of Birmingham, Birmingham B15 2TT, UK; Neuropharmacology Research Group, School of Health Sciences, College of Medicine and Health, University of Birmingham, Birmingham B15 2TT, UK; Department of In Vitro Pharmacology, Celentyx Ltd, Birmingham B15 2SQ, UK; Neuropharmacology Research Group, School of Health Sciences, College of Medicine and Health, University of Birmingham, Birmingham B15 2TT, UK; Department of In Vitro Pharmacology, Celentyx Ltd, Birmingham B15 2SQ, UK; Department of In Vitro Pharmacology, Celentyx Ltd, Birmingham B15 2SQ, UK; Department of In Vitro Pharmacology, Celentyx Ltd, Birmingham B15 2SQ, UK; Department of In Vitro Pharmacology, Celentyx Ltd, Birmingham B15 2SQ, UK; Department of In Vitro Pharmacology, Celentyx Ltd, Birmingham B15 2SQ, UK; Department of In Vitro Pharmacology, Celentyx Ltd, Birmingham B15 2SQ, UK; Department of In Vitro Pharmacology, Celentyx Ltd, Birmingham B15 2SQ, UK; Department of In Vitro Pharmacology, Celentyx Ltd, Birmingham B15 2SQ, UK; Neuropharmacology Research Group, School of Health Sciences, College of Medicine and Health, University of Birmingham, Birmingham B15 2TT, UK; Neuropharmacology Research Group, School of Health Sciences, College of Medicine and Health, University of Birmingham, Birmingham B15 2TT, UK; School of Infection, Inflammation and Immunology, College of Medicine and Health, University of Birmingham, Birmingham B15 2TT, UK; School of Infection, Inflammation and Immunology, College of Medicine and Health, University of Birmingham, Birmingham B15 2TT, UK; Department of Neurosurgery, Queen Elizabeth Hospital Birmingham, Birmingham B15 2QW, UK; NIHR Surgical Reconstruction and Microbiology Research Centre, Queen Elizabeth Hospital Birmingham, Birmingham B15 2GW, UK; School of Infection, Inflammation and Immunology, College of Medicine and Health, University of Birmingham, Birmingham B15 2TT, UK; Department of Neurosurgery, Queen Elizabeth Hospital Birmingham, Birmingham B15 2QW, UK; Department of Cancer and Genomic Sciences, School of Medical Sciences, College of Medicine and Health, University of Birmingham, Birmingham B15 2TT, UK; Department of Neurosurgery, Queen Elizabeth Hospital Birmingham, Birmingham B15 2QW, UK; Department of Neurosurgery, Queen Elizabeth Hospital Birmingham, Birmingham B15 2QW, UK; Department of Metabolism and Systems Science, School of Medical Sciences, College of Medicine and Health, University of Birmingham, Birmingham B15 2TT, UK; Department of Neurosurgery, Queen Elizabeth Hospital Birmingham, Birmingham B15 2QW, UK; Department of Neurosurgery, Queen Elizabeth Hospital Birmingham, Birmingham B15 2QW, UK; Department of Neurosurgery, Queen Elizabeth Hospital Birmingham, Birmingham B15 2QW, UK; Department of Physiology and Medical Physics, RCSI University of Medicine and Health Sciences, Dublin D02 HC66, Ireland; School of Infection, Inflammation and Immunology, College of Medicine and Health, University of Birmingham, Birmingham B15 2TT, UK; Department of Neurosurgery, Queen Elizabeth Hospital Birmingham, Birmingham B15 2QW, UK; NIHR Surgical Reconstruction and Microbiology Research Centre, Queen Elizabeth Hospital Birmingham, Birmingham B15 2GW, UK; Department of Neurosurgery, Queen Elizabeth Hospital Birmingham, Birmingham B15 2QW, UK; Department of Neurosurgery, Queen Elizabeth Hospital Birmingham, Birmingham B15 2QW, UK; Department of Neurosurgery, Queen Elizabeth Hospital Birmingham, Birmingham B15 2QW, UK; School of Infection, Inflammation and Immunology, College of Medicine and Health, University of Birmingham, Birmingham B15 2TT, UK; NIHR Surgical Reconstruction and Microbiology Research Centre, Queen Elizabeth Hospital Birmingham, Birmingham B15 2GW, UK; NIHR Surgical Reconstruction and Microbiology Research Centre, Queen Elizabeth Hospital Birmingham, Birmingham B15 2GW, UK; Department of Neurosurgery, Queen Elizabeth Hospital Birmingham, Birmingham B15 2QW, UK; Department of Psychiatry, University of Oxford, Oxford OX3 7JX, UK; Institute for Mental Health, University of Birmingham, Birmingham B15 2TT, UK; Birmingham Early Intervention Service, Birmingham Women’s and Children’s NHS Foundation Trust, Birmingham B15 2TG, UK; Neuropharmacology Research Group, School of Health Sciences, College of Medicine and Health, University of Birmingham, Birmingham B15 2TT, UK; Department of In Vitro Pharmacology, Celentyx Ltd, Birmingham B15 2SQ, UK; Department of In Vitro Pharmacology, Celentyx Ltd, Birmingham B15 2SQ, UK; Neuropharmacology Research Group, School of Health Sciences, College of Medicine and Health, University of Birmingham, Birmingham B15 2TT, UK; Department of In Vitro Pharmacology, Celentyx Ltd, Birmingham B15 2SQ, UK; School of Infection, Inflammation and Immunology, College of Medicine and Health, University of Birmingham, Birmingham B15 2TT, UK; Department of Neurosurgery, Queen Elizabeth Hospital Birmingham, Birmingham B15 2QW, UK; NIHR Surgical Reconstruction and Microbiology Research Centre, Queen Elizabeth Hospital Birmingham, Birmingham B15 2GW, UK; Neuropharmacology Research Group, School of Health Sciences, College of Medicine and Health, University of Birmingham, Birmingham B15 2TT, UK; NIHR Surgical Reconstruction and Microbiology Research Centre, Queen Elizabeth Hospital Birmingham, Birmingham B15 2GW, UK

**Keywords:** P2X7 receptor, microglia, traumatic brain injury, neuroinflammation, P2X7 receptor antagonists, NLRP3 inflammasome inhibitors

## Abstract

Traumatic brain injury is a major cause of death throughout the world, and currently there are no approved drugs to treat this debilitating condition, emphasizing the clear unmet substantial clinical need. It is well recognized that microglial activation and the hostile neuroinflammatory response arising after the initial insult provide a therapeutic window for pharmacological intervention. The purinergic P2X7 receptor (P2X7R) is a key driver of neuroinflammation in a range of animal models of traumatic brain injury.

To generate translational evidence for the role of the P2X7R, we optimized two human inflammatory models, human cells (monocyte-derived microglia) *in vitro* and human brain tissue *ex vivo*, to test the impact of clinical-stage brain-penetrant P2X7R antagonists.

Using lipopolysaccharide-primed human monocyte-derived microglia, the P2X7R agonist 2′(3′)-*O*-(4-benzoylbenzoyl) ATP (BzATP), evoked a concentration-dependent increase in pro-inflammatory interleukin (IL)-1β and IL-18 release, which was antagonized in a concentration-dependent manner by selective P2X7R antagonists and also by inhibitors of either the NLRP3 complex or caspase-1, implicating a role for the inflammasome in P2X7R-mediated cytokine release. Using slices of human brain tissue, BzATP similarly evoked cytokine release in a concentration-dependent manner that was also antagonized by selective P2X7R antagonists at pharmacologically relevant concentrations.

The present study demonstrates the ability of P2X7R antagonists to suppress the neuroinflammatory response from primed human monocyte-derived microglia and human brain slices to offer direct translational data that central P2X7R antagonism might limit pathology-driven pro-inflammatory responses in the brain. This is predicted to improve the clinical outcomes for patients with traumatic brain injury and other neuroinflammatory pathologies.

## Teaser

Cowley *et al.* show that CNS-penetrant, selective P2X7 receptor antagonists reduce the release of pro-inflammatory cytokines from human microglia and brain tissue. These findings support P2X7 receptor antagonism as a potential therapeutic strategy to improve outcomes following traumatic brain injury.

## Introduction

Traumatic brain injury (TBI) is a global health problem and is the leading cause of death and disability in the first four decades of life.^1^ In addition to the serious impact upon patients and their families, estimated total costs in the USA alone exceed US$40 billion per annum.^2-4^ Current clinical management focuses on the maintenance of life and early detection of associated secondary injuries, because the damage associated with primary injury is often irreversible.^5,6^ Nevertheless, despite the evident major clinical need, there are currently no approved pharmacological treatments to improve clinical outcomes for patients with TBI.^1,7,8^

The mechanical damage associated with TBI drives a neuroinflammatory response that triggers a cascade of disturbances to CNS homeostasis, such as ionic imbalance,^9,10^ glutamate excitotoxicity,^11,12^ oxidative stress,^13^ lipid peroxidation,^14^ neuronal apoptosis^15^ and mitochondrial dysfunction.^16,17^ The primary response to the injured site is characterized by activation of CNS-resident microglia^18-23^ and astrocytes.^24,25^ In animal models, depletion of microglia is sufficient to reverse neuroinflammation and neuropathology.^20,26^ Activated microglia and astrocytes can secrete chemokines^27^ that promote the infiltration of circulating immune cells, such as monocytes,^28,29^ macrophages^30,31^ and neutrophils.^32^ This facilitates a local pro-inflammatory environment and oedema, with consequential damage to the integrity of the blood–brain barrier (BBB),^33^ facilitating further entry of peripheral immune cells.^34^ The introduction of sampling from the injured brain using microdialysis has seen a step-change in the quality of relevant data to inform pathological cellular mechanisms.^35^ It is now better understood that the neuroinflammatory response is triggered almost immediately post-injury and can persist for months.^36-39^ Upregulation of cytokines, such as pro-inflammatory interleukin (IL)-1β, occurs within hours of TBI in both rodent and human studies.^36,37,40-42^ This pro-inflammatory response instigates an increasing cycle of pathological processes^43^ that probably contribute to the secondary injury, increasing the volume of brain tissue damaged by the initial TBI into the penumbra,^44^ to the further detriment of the patient’s clinical outcomes and subsequent quality of life.^45,46^ Pharmacological intervention to limit the damage associated with this latter secondary injury is likely to offer the greatest potential to improve the clinical outcomes for patients.^45^

The purinergic P2X7 receptor (P2X7R) is a central driver of inflammation and is activated by millimolar concentrations of ATP.^47^ The P2X7R is expressed by microglia,^48-54^ the resident immune cells of the CNS,^55,56^ that participate in a multitude of developmental, homeostatic and pathological roles.^57-60^ Activation of the P2X7 ligand-gated ion channel receptor triggers cation influx and potassium efflux,^61^ leading to activation of the NLRP3 inflammasome^62^ and the subsequent processing and release of the pro-inflammatory cytokines IL-1β and IL-18.^63-74^ Animal models of TBI are associated with a substantial cellular efflux of the endogenous purinergic agonist ATP into the extracellular space^54,75^ sufficient to activate the P2X7R, such that antagonists reduce arising neuronal damage and pro-inflammatory cytokine release in rodent models of neuroinflammation,^64,76-78^ neurotrauma^54,79-81^ and neuropathic pain.^82^ If the data from animal models translate to the clinical situation, following TBI the bathing of the penumbra in high concentrations of extracellular ATP discharged from the damaged tissue would result in P2X7R activation. This would suggest that P2X7R antagonists would limit neuroinflammatory and secondary damage, which warrants further investigation in the prospect of finally identifying an efficacious pharmacological therapy for TBI. Reported clinical studies to date with Pfizer’s selective P2X7R antagonist, CE-224,535,^83^ include peripheral inflammatory conditions, such as arthritis, and despite the medication being well tolerated, clear overall therapeutic benefit was not apparent,^84^ although there were issues with the trial design. CE-224,535 appears to penetrate the intact BBB poorly,^85^ a hurdle that has been overcome by clinical stage selective P2X7R antagonists, such as JNJ-54175446 and JNJ-55308942, that are likely to be more amenable to impact central neuroinflammatory disorders,^86-92^ which further highlights the therapeutic opportunity in TBI and beyond.

Critical to the clinical translation of P2X7R antagonists, major pharmacological differences exist between the mouse, rat and human P2X7R,^93,94^ and important differences exist between rodent and human microglia relevant to their pro-inflammatory response and disease-relevant gene expression.^95,96^ Human primary microglia are embedded deep within CNS tissue, making their isolation difficult, and efforts to mitigate their activation to date have often failed.^97^ Even once isolated from the CNS, further issues arise owing to their restricted proliferative capacity in culture,^98^ and concerns remain around phenotypic changes to their CNS identity once removed from their brain microenvironment^97,99^ or following antemortem and post-mortem delay.^100^ Considerable recent efforts have focused on induced pluripotent stem cell-derived microglia, which offer a suitable model to study inflammation, although some limitations are apparent.^101,102^ In the present study, we used an alternative human microglia model, in which human peripheral blood monocytes are differentiated into microglia-like cells, so-called human monocyte-derived microglia (hMDM), a system that has been increasingly shown to be informative to study a range of neuroinflammatory/neurodegenerative diseases.^103-112^ This enabled us to demonstrate the ability of clinically relevant P2X7R antagonists, including brain-penetrant molecules, to curtail pro-inflammatory cytokine release. The relevance of these findings was then further translated using adult human precision-cut brain slices generated subsequent to neurosurgical resections for clinical management. This latter tissue preparation better reflects the functional interconnections of different cell types in the brain, which may also express the P2X7R.^113^ Our findings provide direct relevant evidence for the use of P2X7R antagonism to inhibit human microglia-mediated inflammation, with arising potential benefits for patients with TBI and other neuroinflammatory diseases.

## Materials and methods

### Generation of human monocyte-derived microglia

Peripheral blood mononuclear cells were isolated from leucocyte cones (from healthy donors; National Health Service Blood and Transplant (NHS-BT) through Ficoll-Paque PLUS (GE Healthcare) density centrifugation, and monocytes were further enriched by negative selection using EasySep Human Monocyte Enrichment Kits (STEMCELL Technologies). Anti-CD14 (BioLegend) labelling was used to verify CD14^+^ monocyte purity. For differentiation into hMDM, monocytes were cultured [black-walled imaging plates (Greiner)] at 1.0 × 10^6^/ml in serum-free RPMI-1640 with penicillin/streptomycin (penicillin 100 U and streptomycin 0.1 mg/ml; Sigma), plus human (h) hM-CSF, hGM-CSF, hNGF-β, hCCL2 and hIL-34 (BioLegend) for 5–7 days in an atmosphere of air enriched with 5% CO_2_ at 37°C.

### Immunolabelling of human monocyte-derived microglia

For immunocytochemistry, hMDM and originating monocytes were fixed with 4% paraformaldehyde (PFA) for 12 min and washed twice with PBS (Gibco). Cells were permeabilized with 0.1% Triton X-100 (Sigma) for 10 min, blocked with 5% goat serum (Sigma) in PBS + 0.1% Tween-20 (Sigma) for 1 h and labelled with primary antibodies at room temperature for 1 h. After washing with PBS (Sigma), cells were incubated with secondary antibodies at room temperature in the dark for 1 h. Primary antibodies used in this study were anti-TMEM119 (ATLAS; 1:250), anti-P2RY12 (ATLAS; 1:250), anti-CX3CR1 (Invitrogen; 1:100), anti-TREM2 (Invitrogen; 1:200), anti-Iba1 (Invitrogen; 1:300), anti-P2X7 (DSHB; 1:100), anti-CD45 (BioLegend; 1:250) and anti-CD68 (BioLegend; 1:200) diluted in blocking solution. Depending on the species of the primary antibodies, either Alexa Fluor 488 (Invitrogen; 1:500) or Alexa Fluor 647 (Invitrogen; 1:500) conjugated secondary antibodies were used, and cell nuclei were stained with Hoechst 33342 (Thermo Fisher; 1:1000). Cells were imaged and immunofluorescence was quantified using high-content confocal imaging (Yokogawa CQ1 imaging platform; 40× objective lens, imaging at least nine fields of view per well). Positive cell surface immunolabelling on cells was quantified as the total intensity per cell, using CQ1 image analysis software (version 1.02.02.00).

### Human monocyte-derived microglia cytokine release assays

After 5–7 days of culture, hMDM were primed with lipopolysaccharide (LPS; 100 ng/ml; InvivoGen) for 2 h, followed by, where relevant, addition of antagonists [JNJ-54175446 or JNJ-55308942 both (generous gifts from Janssen) or A-804598 (Sigma)] or relevant vehicle (DMSO; 0.03%) for 30 min prior to 4 h treatment with 2′(3′)-*O*-(4-benzoylbenzoyl) ATP (BzATP; Sigma) or vehicle (water; 0.14%). For experiments investigating inflammasome pathway modulation, hMDM were preincubated with the selective inflammasome inhibitor MCC950 (Selleck Chemicals) or the selective caspase-1 inhibitor VX-765 (InvivoGen) or relevant vehicle (water/DMSO; 0.03%) for 1 h prior to priming with LPS (100 ng/ml), for 2 h, followed by stimulation with BzATP for 4 h.

### Human brain slice culture experiments

Human brain tissue, obtained upon resection from the cerebral cortex or cerebellar cortex (patient demographics are summarized in Supplementary Table 1), was placed immediately in ice-cold transport medium (Hibernate-A; Gibco) and processed within 20–30 min at 4°C using a McIlwain chopper (Stoelting) to yield slices of an optimal size (350 µm × 350 µm × thickness of brain tissue). The slices were washed twice with ice-cold Hanks' balanced salt solution (HBSS; Gibco), and approximately equal numbers of slices were plated into each well of a 96-well plate and rested overnight at 37°C, 5% CO_2_ in Neurobasal-A (Gibco; 10888022) medium containing penicillin/streptomycin (penicillin 100 U and streptomycin 0.1 mg/ml; Sigma), supplemented with 2% B27 (Gibco).

### Human brain slice cytokine release assays

After resting overnight, human brain slices were washed twice with pre-warmed (37°C) HBSS and resuspended in RPMI-1640 supplemented with 1% B27. Where stated, slices were primed with LPS (100 ng/ml; InvivoGen) for 2 h, followed by where relevant, addition of the antagonist A-804598 (Sigma) or relevant vehicle (DMSO; 0.03%) for 30 min prior to 4 or 18 h treatment with BzATP (Sigma) or vehicle (water; 0.14%). For studies investigating antagonism of the BzATP response by JNJ-54175446, the brain slices were not primed with LPS.

### Assay of biomarkers

Supernatants from cultures of hMDM and human brain slices were harvested, centrifuged at 400*g* for 5 min and stored at −80°C until analysis. Biomarkers in the tissue culture supernatants were quantified by ELISA (IL-1β and IL-18; R&D Systems) or the Luminex platform (ProcartaPlex Human Cytokine/Chemokine/Growth Factor convenience Panel; Thermo Fisher) according to the manufacturer’s instructions. Sample acquisition was performed in triplicates using a Bio-Rad Bio-Plex BP-200 instrument.

### Immunolabelling of human dissociated brain tissue

Human brain tissue (as earlier) was initially minced into approximately 3 mm × 3 mm slices before incomplete digestion with hyaluronidase, collagenase and DNAse (7.5 mg/ml, 1.0 mg/ml and 40 μg/ml, respectively; all Sigma) for 90 min at 37°C. The cell suspension was then washed through a 100 μm cell strainer with complete Dulbecco’s modified Eagle medium/F-12 medium (Gibco). Arising cells were plated at 2.0 × 10^6^/ml in imaging plates (Greiner) and allowed to rest overnight in complete medium. Non-adhered cells were then removed with wash steps and adherent cells fixed with 4% PFA, washed with PBS and permeabilized with 0.1% Triton X-100 (Sigma) for 10 min, followed by blocking with 5% goat serum (Sigma) in PBS + 0.1% Tween-20 (Sigma) for 1 h. Cells were then immunolabelled with anti-P2X7R (DSHB), anti-TMEM119 (ATLAS) and anti-P2RY12 (ATLAS) antibodies, followed by visualization via Alexa Fluor 488 (Invitrogen) or Alexa Fluor 647 (Invitrogen) secondary antibodies for 1 h at room temperature in the dark.

### Study approval

All human tissue samples were obtained with written informed consent and with approval from the appropriate Research Ethics Committee (brain tissue; REC reference 20/WA/0327; UHB Research Project Authorization RRK732). Human fresh blood cells were obtained from NNHS-BT with approval from an appropriate research ethics committee (REC reference 20/WA/0216). All research was carried out in accordance with relevant guidelines and regulations. All methods were approved by the relevant institutions.

### Statistical analysis

Data are presented as the mean + (or ±) standard error of the mean (SEM) from at least five independent experimental repeats (independent repeats used cells/tissue from a different donor) unless otherwise indicated. Differences among multiple treatment groups were determined by a one-way ANOVA test, and differences between two groups only were determined using Student’s unpaired *t*-test. Where data were non-parametric, Kruskal–Wallis and Wilcoxon signed-rank tests were used. Normality was determined using the Shapiro–Wilk test. The specific analysis used is indicated in the figure legend. Values of *P* < 0.05 were considered statistically significant. The *P*-values are indicated with an asterisk or a plus symbol on each graph (*^/+^*P* < 0.05, **^/++^*P* < 0.01 and ***^/+++^*P* < 0.001) and are shown in the figures. Iterative curve-fitting data analysis using, where appropriate, a four-parameter logistic equation was performed using KaleidaGraph (version 4.5.4, 2018; Synergy Software, Reading, PA, USA) and GraphPad Prism Software (version 10.0.3; GraphPad Prism Software LLC, Boston, MA, USA). All statistical analysis and generation of graphs was performed in GraphPad Prism Software (version 10.0.3; GaphPad Software LLC, Boston, MA, USA) unless otherwise stated.

## Results

### Phenotypic characterization of human monocyte-derived microglia

Microglia, being essentially specialized macrophages of the CNS, share various common markers with peripheral macrophages, including ionized calcium binding receptor 1 (Iba1), fractalkine receptor (CX3CR1) and triggering receptor expressed on myeloid cells 2 (TREM2), whereas transmembrane protein 119 (TMEM119) and purinergic receptor (P2RY12) expressions are often considered as specific phenotypic markers of CNS-resident microglia. Initial experiments in this study used both myeloid and microglia-specific markers to characterize our hMDM against originating monocytes and GM-CSF + IL-34-only treated cells. After 5–7 days in culture, hMDM displayed typical microglia-like characteristics, such as a small soma and elongated morphology, in comparison to the rounded appearance of originating monocytes (Fig. 1A). As assessed using immunocytochemistry and quantified by total fluorescence intensity per cell (Fig. 1B and C), TMEM119 and P2RY12 expression was relatively high on hMDM [1.91 ± 0.10 × 10^6^ and 1.62 ± 0.19 × 10^6^ RFU (relative fluorescent units)], with both markers appearing lower on originating monocytes (6.91 ± 1.99 × 10^5^ and 8.34 ± 1.24 × 10^5^ RFU) and GM-CSF + IL-34-treated cells (1.30 ± 0.09 × 10^6^ and 8.80 ± 1.67 × 10^5^ RFU; Fig. 1B). In addition, hMDM also expressed common myeloid markers known to be upregulated by activated microglia, such as CX3CR1, TREM2 and Iba1 (Fig. 1B and C). These markers were present ubiquitously on all three cell types, although notably expression was highest on hMDM (3.59 ± 0.41 × 10^6^, 1.46 ± 0.15 × 10^6^ and 1.33 ± 0.18 × 10^7^ RFU; Fig. 1B) compared with originating monocytes (1.69 ± 0.25 × 10^6^, 8.21 ± 1.55 × 10^5^ and 4.73 ± 0.79 × 10^6^ RFU; Fig. 1B). The P2X7R was readily expressed across all three cell types (Fig. 1B and C). Monocytes evidently displayed higher CD68 labelling (2.57 ± 0.26 × 10^6^ RFU) than hMDM (9.59 ± 1.35 × 10^5^ RFU) and GM-CSF + IL-34-treated cells (4.67 ± 0.40 × 10^5^ RFU; Fig. 1B), whilst CD45 expression was higher on hMDM and GM-CSF + IL-34-treated cells (1.21 ± 0.89 × 10^6^ and 1.14 ± 0.69 × 10^6^ RFU) than on monocytes (0.75 ± 0.10 × 10^6^ RFU) (Fig. 1B and C). Together, these results confirm that hMDM express key markers enriched in human brain microglia and are phenotypically distinct from originating monocytes.

**Figure 1:**
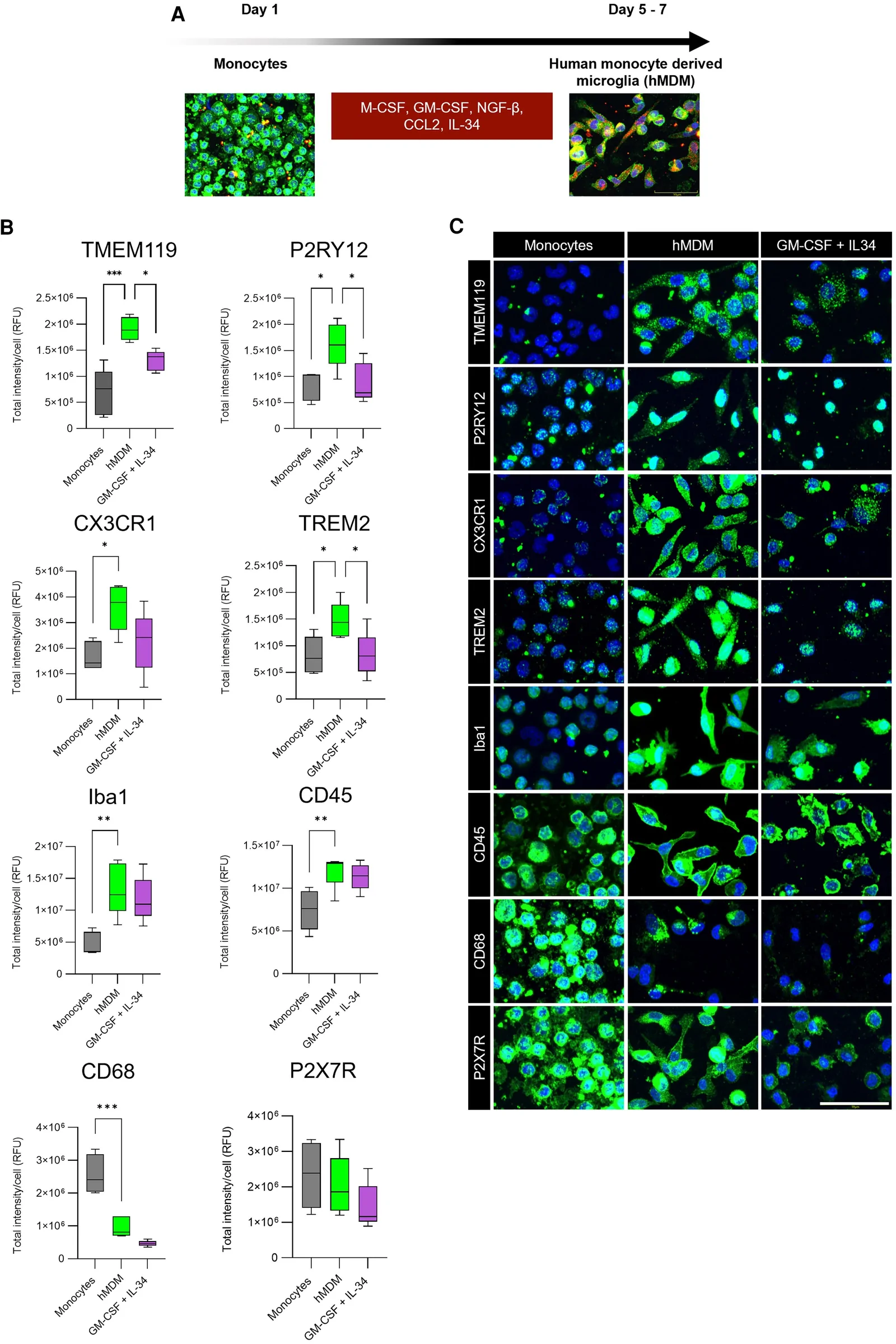
**Generation and characterization of human monocyte-derived microglia.** (**A**) Differentiation of peripheral blood monocytes to hMDM over 5–7 days in culture using a cocktail of cytokines, with representative confocal images displaying co-immunolabelling of the P2X7 receptor (green) and TMEM119 (red) and with cell nuclei stained with Hoechst 33342 (blue). (**B**) Quantification of immunostaining total intensity [as relative fluorescent units (RFU)] for monocytes (grey-filled bars), hMDM (green-filled bars) and GM-CSF + IL-34-treated cells (purple-filled bars) from images acquired by high-content confocal microscopy for cells labelled in **C**. (**C**) Representative confocal images of hMDM and originating monocytes displaying immunolabelling of TMEM119, P2YR12, CX3CR1, TREM2, Iba1, CD45, CD68 and P2XR7 (green), with cell nuclei stained with Hoechst 33342 (blue), (original magnification, ×40). Images are maximum-intensity projections from sequential *z*-stacks. Scale bar = 50 μm. Data are expressed as the mean ± standard error of the mean from five independent experiments with cells originating from five different donors. Significance was determined using a one-way ANOVA with Tukey’s *post hoc* test for comparison of phenotypic marker expression between monocytes, hMDM and GM-CSF + IL-34-treated cells. Significant difference between indicated data: *P < 0.05, **P < 0.01 and ***P < 0.001. GM-CSF = granulocyte-macrophage colony-stimulating factor; hMDM = human monocyte-derived microglia; IL = interleukin.

### P2X7R-dependent soluble factor release from human monocyte-derived microglia

Next, we identified the functional capacity of hMDM to respond to P2X7R activation using a range of soluble factors as the readout. Initially, we investigated the concentration-dependent impact of the non-selective P2X7R agonist BzATP on LPS-primed hMDM IL-1β and IL-18 cytokine release. BzATP induced a concentration-dependent release of IL-1β and IL-18 in LPS-primed hMDM cultures (IL-1β, pEC_50_ = 4.04 ± 0.05; and IL-18, pEC_50_ = 3.95 ± 0.02, respectively; Fig. 2 and Supplementary Table 2). To characterize the P2X7R-evoked cytokine release by hMDM further, LPS-primed hMDM cultures were treated with a near-maximal effective concentration of BzATP, and the release of 45 different cytokines, chemokines and growth factors was quantified using a ProcartaPlex kit (Fig. 3; values are in picograms per millilitre; see Supplementary Table 3). The greatest mean LPS + BzATP-treatment percentage change relative to basal release levels was for IL-1β (19 817% ± 3152%), followed by IL-6 (14 002% ± 6663%), IL-1α (3908% ± 1093%), TNF-α (1665% ± 645%), MIP-1β (942% ± 615%) and, lastly, IL-18 (556% ± 67%) (Fig. 3A–C). Notably, the BzATP-evoked release of IL-1β, IL-1α and IL-18 was antagonized by pretreatment with the moderate-affinity and selective competitive P2X7R antagonist, A-804598 (tested at 3.0 μM; IL-1β, 1017% ± 216%; IL-1α, 299% ± 82%; and IL-18, 153% ± 11%; Fig. 3A–C). As expected, various other cytokines and chemokines displayed LPS-dependent release, e.g. TNF-α, IL-6, IL-10, MIP-1-β, IL-23 and FGF-2 (4163% ± 1340%, 47 362% ± 34 780%, 2238% ± 1319%, 2084% ± 975%, 599% ± 237% and 491% ± 31%, respectively), although a consistent impact of BzATP addition was not apparent with these latter soluble factors (Fig. 3A–C).

**Figure 2:**
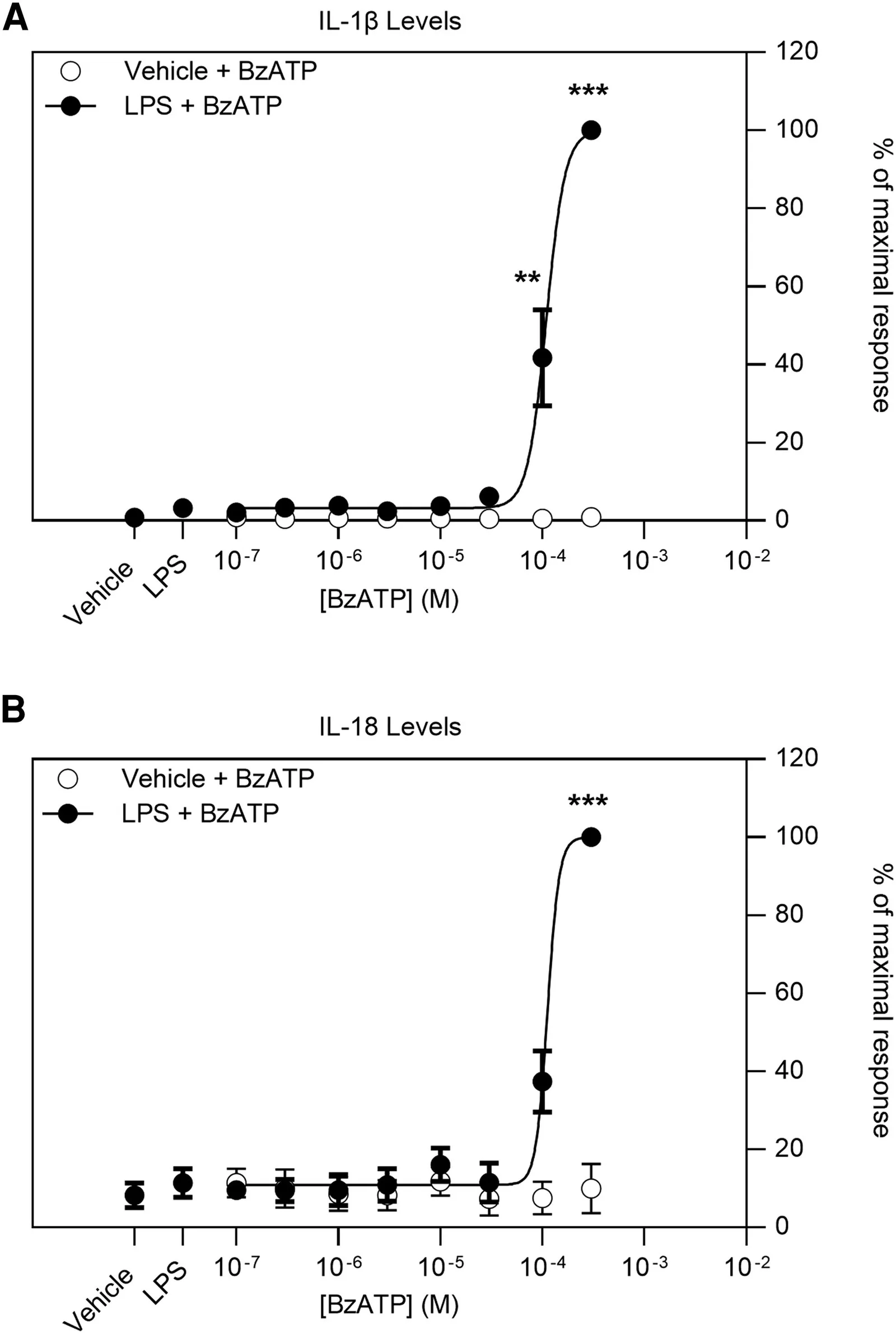
**Concentration-dependent BzATP-induced IL-1β and IL-18 release from LPS-primed hMDM.** Human monocyte-derived microglia (hMDM) cultures were treated in the absence (vehicle) or presence of LPS (100 ng/ml; 2 h) and were subsequently stimulated with the non-selective P2X7 receptor agonist BzATP (0.3–300 μM) for 4 h when release of IL-1β (**A**) and IL-18 (**B**) was monitored. Graphs display percentage release relative to a maximal response at 300 μM BzATP and represent an iterative curve fit according to a four-parameter logistic equation. Data are expressed as the mean ± standard error of the mean from five independent donors. Significance was determined using a one-way ANOVA with Dunnett’s *post hoc* test for comparison of maximal picograms per millilitre BzATP-induced IL-1β release (300 µM BzATP; IL-1β levels = 2688 ± 134 pg/ml and IL-18 levels = 282 ± 56 pg/ml) compared with either LPS (IL-1β levels = 2.43 ± 0.58 pg/ml and IL-18 levels = 11.35 ± 3.67 pg/ml) or vehicle control (IL-1β levels = 1.02 ± 0.23 pg/ml and IL-18 levels = 8.21 ± 3.19 pg/ml). Significant difference between indicated data: **P < 0.01 and ***P < 0.001. BzATP = 2′(3′)-*O*-(4-benzoylbenzoyl) ATP; IL = interleukin; LPS = lipopolysaccharide.

**Figure 3:**
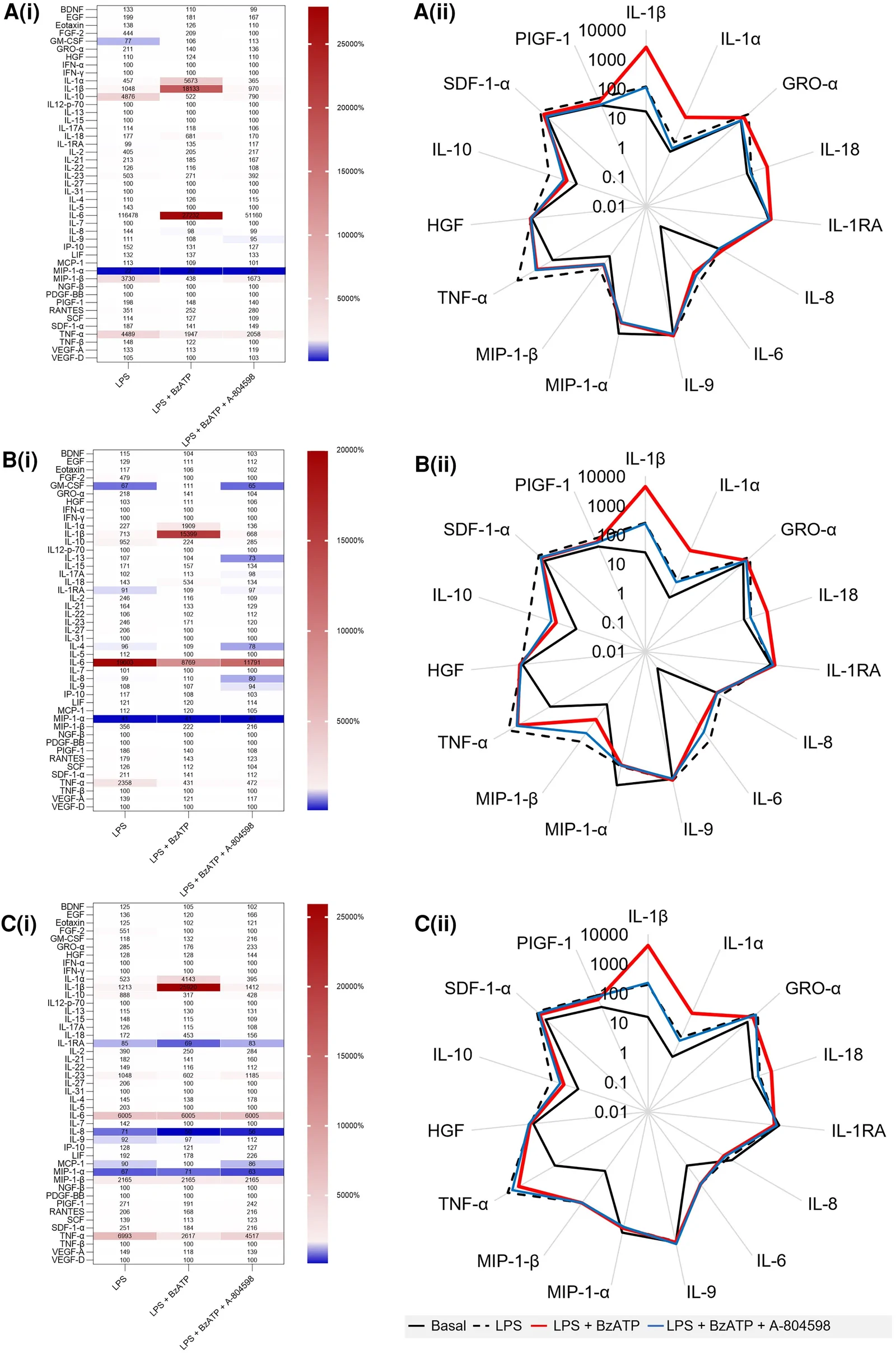
**P2X7 receptor-dependent release of various biochemicals from LPS-primed hMDM.** Human monocyte-derived microglia (hMDM) cultures were primed with LPS (100 ng/ml), with subsequent addition of the non-selective P2X7 receptor agonist BzATP (100 μM) in the absence (vehicle) or presence of the selective P2X7 receptor antagonist, A-804598 (3.0 μM). Heat maps [**A**(**i**), **B**(**i**) and **C**(**i**)] show percentage fold change in cytokine levels relative to vehicle (i.e. no LPS priming), and radar plots [**A**(**ii**), **B**(**ii**) and **C**(**ii**)] show cytokine release in picograms per millilitre (except for IL-1RA, IL-6, IL-8 and MIP-1-β levels shown as nanograms per millilitre) from cultures treated with vehicle (black solid line), LPS (black dashed line), LPS + BzATP (red solid line) and LPS + BzATP + A-804598 (blue solid line). Data show three independent donors {Donor 1 [**A**(**i** and **ii**)], Donor 2 [**B**(**i** and **ii**)] and Donor 3 [**C**(**i** and **ii**)]} expressed as the mean percentage fold change derived from triplicate wells. BzATP = 2′(3′)-*O*-(4-benzoylbenzoyl) ATP; IL = interleukin; LPS = lipopolysaccharide; MIP = macrophage inflammatory protein.

In addition to releasing a plethora of soluble factors, hMDM readily phagocytosed human myelin basic protein, and uptake was amendable to inhibition with the actin polymerization dysrupter, cytochalasin-D (Supplementary Fig. 1), confirming that these cells are capable of performing crucial microglial functions. In addition to key phenotypic markers, hMDM also express HLA-DR (Supplementary Fig. 2), implying that hMDM might also function as antigen presenting cells. We also investigated the impact of BzATP stimulation (using either LPS-primed or non-primed hMDM), on the expression of the P2X7R and the homeostatic marker, Iba1, by immunocytochemistry (Supplementary Fig. 3). No significant impact of BzATP was evident on Iba1 expression (Supplementary Fig. 3B), whilst a small, but non-significant reduction in P2X7R expression was apparent during BzATP stimulation of both LPS-primed and non-LPS-primed hMDM cultures (Supplementary Fig. 3A). Further to this, we also investigated the impact of P2X7R activation upon hMDM morphology, noting little impact upon cellular circularity (Supplementary Fig. 3C) and cellular circumference (Supplementary Fig. 3D).

Together, these results confirm the expression of human functional P2X7Rs by hMDM that promote pro-inflammatory IL-1β and IL-18 release and demonstrate that the assay window is suitable for studying modulators of this response.

### P2X7R inhibition by clinical-stage P2X7R antagonists

The hMDM model was also used to investigate the pharmacology of two clinical stage competitive P2X7R antagonists that penetrate the BBB readily, JNJ-54175446 and JNJ-55308942, in comparison to another P2X7R antagonist, A-804598. JNJ-54175446 and JNJ-55308942, along with A-804598, displayed concentration-dependent antagonism of a near-maximal effective concentration of BzATP to evoke IL-1β cytokine release from LPS-primed hMDM; maximal antagonism of the BzATP-induced response was evident for all three compounds (Fig. 4A–C). Of the three antagonists investigated, the rank order of apparent affinity (pIC_50_) to prevent BzATP-induced IL-1β release was JNJ-54175446 (pIC_50_ = 8.92 ± 0.14) > JNJ-55308942 (pIC_50_ = 8.18 ± 0.08) ≥ A-804598 (pIC_50_ = 8.09 ± 0.25) (Fig. 4A–C and Supplementary Table 2). The ability of these three antagonists to inhibit the BzATP-induced increase in IL-18 displayed the same pharmacological rank order of apparent affinity with effective concentrations also in the nanomolar range (Fig. 4D–F and Supplementary Table 2). These data confirm that selective P2X7R antagonists, including one with demonstrated CNS penetrance (JNJ-54175446), mediate potent inhibition of human microglial P2X7-driven pro-inflammatory IL-1β and IL-18 release.

**Figure 4:**
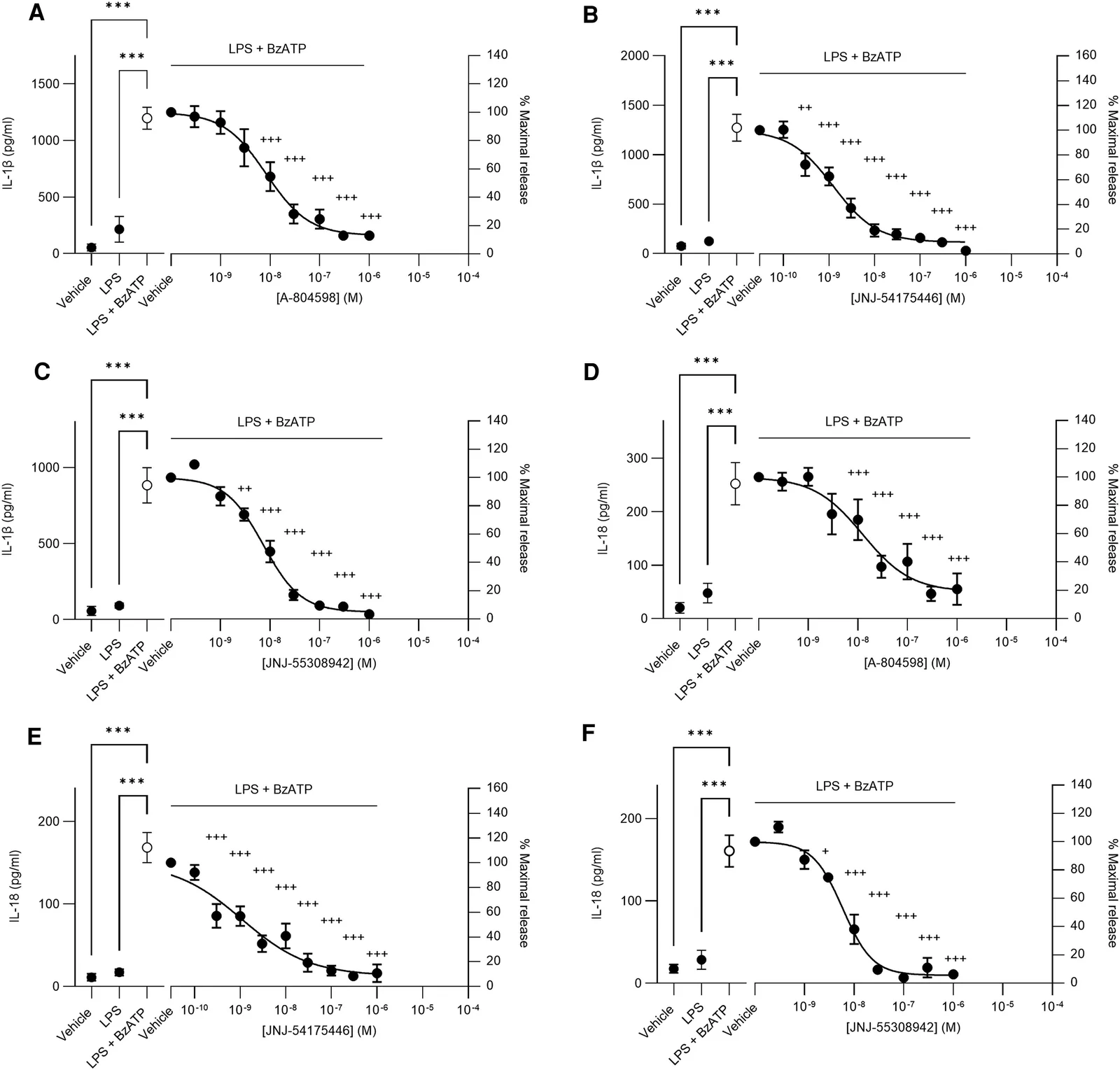
**Ability of various P2X7 receptor antagonists to antagonize BzATP-induced IL-1β and IL-18 release from LPS-primed hMDM.** Human monocyte-derived microglia (hMDM) cultures were treated in the absence (vehicle) or presence of LPS (100 ng/ml) for 2 h and were then incubated for 0.5 h with a P2X7 receptor antagonist [A-804598 (**A** and **D**), JNJ-54175446 (**B** and **E**) or JNJ-55308942 (**C** and **F**)], followed by stimulation with BzATP (100 μM) for 4 h. Filled circles (*left y*-axis) display IL-1β (**A**–**C**) or IL-18 levels (**D**–**F**) in the cell culture supernatants (in picograms per millilitre) and curve fits (iterative curve fit according to a four-parameter logistic equation) of the impact of the indicated P2X7 receptor antagonist upon percentage release relative to the LPS + BzATP response (open circles; 100 µM BzATP). Data are expressed as the mean ± standard error of the mean from at least five independent donors. Significance was determined using a one-way ANOVA with Dunnett’s *post hoc* test for comparison of picograms per millilitre BzATP-induced IL-1β or IL-18 release compared with either LPS or vehicle control (*left y*-axis), or comparison of percentage IL-1β release at each concentration of P2X7 receptor antagonist to vehicle control (set to 100%; *right y-*axis). Significant difference between indicated data: ***P < 0.001. Significant difference from LPS + BzATP: ^+^*P <* 0.05, ^++^*P <* 0.01 and ^+++^*P <* 0.001. BzATP = 2′(3′)-*O*-(4-benzoylbenzoyl) ATP; IL = interleukin; LPS = lipopolysaccharide.

### Modulation of inflammasome pathway-mediated IL-1β and IL-18 release

We extended the study with the hMDM model to investigate the impact of the selective NLRP3 inflammasome inhibitor MCC950 and the caspase-1 inhibitor VX-765 upon BzATP-evoked release of IL-1β and IL-18. Either MCC950 or VX-765 was effective, in a concentration-dependent manner, to inhibit BzATP-evoked IL-1β and IL-18 release (Fig. 5A–D and Supplementary Table 2); MCC950 was more potent than VX-765 (Fig. 5A and B and Supplementary Table 2), although, notably, near-maximal inhibition was not evident with VX-765-treated cell cultures at the highest concentration tested (Fig. 5C and D).

**Figure 5:**
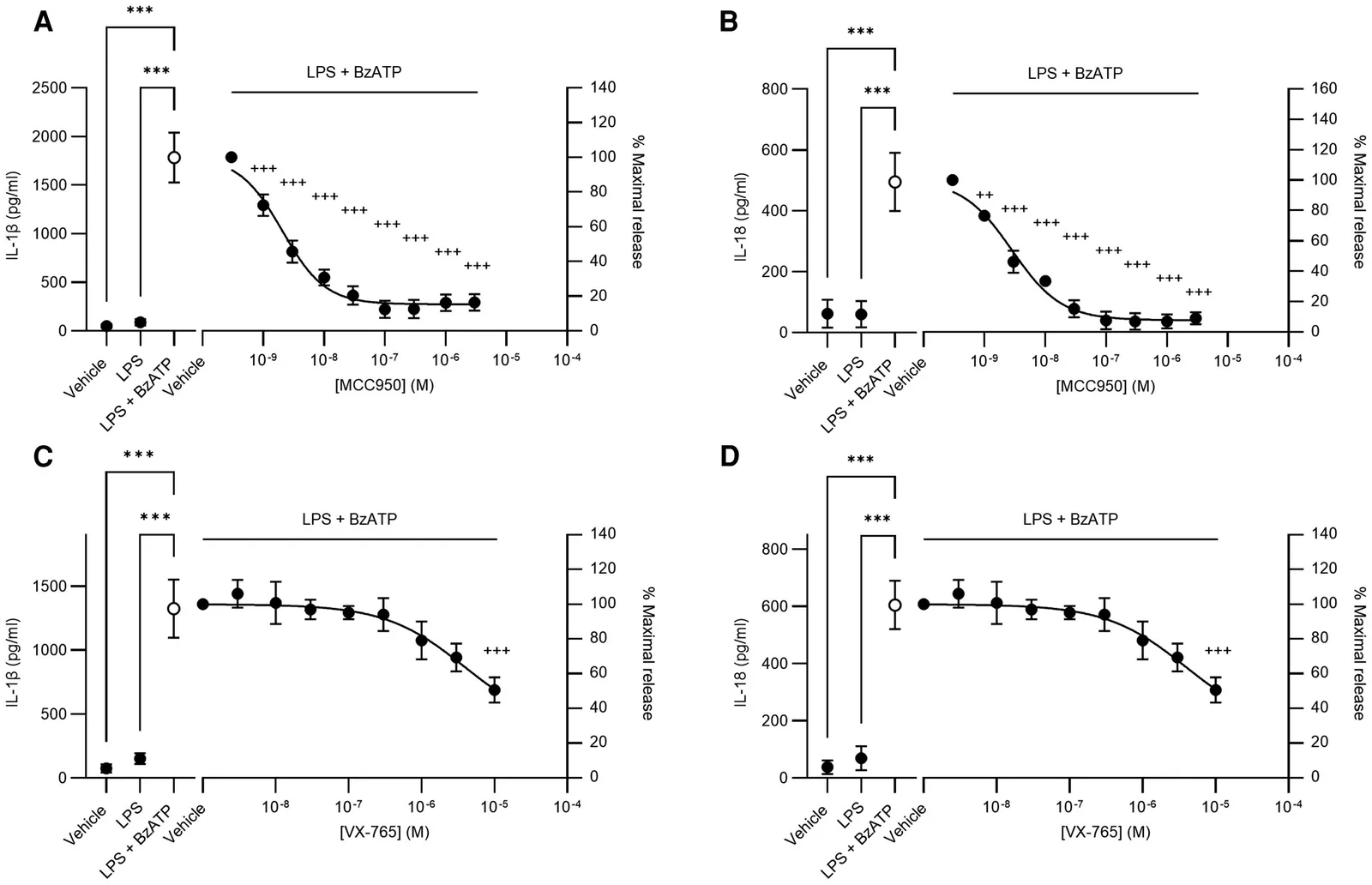
**Ability of inhibitors of the NLRP3 inflammasome or caspase-1 to inhibit BzATP-induced IL-1β and IL-18 release from LPS-primed hMDM.** Human monocyte-derived microglia (hMDM) cultures were preincubated for 1 h in the absence (vehicle) or presence of the NLRP3 inflammasome inhibitor MCC950 (**A** and **B**) or the caspase-1 inhibitor VX-765 (**C** and **D**) prior to priming with LPS (100 ng/ml; 2 h), followed by stimulation with BzATP (100 μM) for 4 h. (**A** and **D**) Three circles to the left (*left y*-axis; mean ± SEM) display IL-1β or IL-18 levels in the cell culture supernatants and curve fits (iterative curve fit according to a four-parameter logistic equation) of the impact of the MCC950 or VX-765 upon percentage release relative to the LPS + BzATP response (open circles; 100 µM BzATP). Data are expressed as the mean ± standard error of the mean from five independent donors. Significance was determined using a one-way ANOVA with Dunnett’s *post hoc* test for comparison of picograms per millilitre BzATP-induced IL-1β or IL-18 release compared with either LPS or vehicle control (*left y*-axis), or for comparison of the percentage IL-1β or IL-18 release at each concentration of inhibitor to their relative vehicle control (set to 100%; *right y*-axis). Significant difference between indicated data: ***P < 0.001. Significant difference from LPS + BzATP: ^++^*P <* 0.01 and ^+++^*P <* 0.001. BzATP = 2′(3′)-*O*-(4-benzoylbenzoyl) ATP; IL = interleukin; LPS = lipopolysaccharide.

### P2X7R-dependent cytokine release from human brain slices

To translate our key findings from hMDM cultures further, we developed a human brain optimized slice culture model using tissue from neurosurgical resections to investigate the ability of the P2X7R expressed on human microglia to drive pro-inflammatory cytokine release. Using a human dissociated glial cell preparation, we confirmed co-expression of the P2X7R with key microglial markers, TMEM119 and P2RY12 (Fig. 6A). In accordance with the findings with the hMDM model, human LPS-treated brain slices demonstrated increased release of IL-1β (Fig. 6B), which was further enhanced in a concentration-dependent manner by BzATP (Fig. 6B and Supplementary Table 4) with comparable potency to that evident using the hMDM model (Fig. 2 and Supplementary Table 2). To extend the human translational evidence for the potential of P2X7R antagonists to suppress inflammatory cytokine release, we demonstrated that the selective P2X7R antagonist A-804598 inhibited a near-maximal effective concentration of BzATP to evoke IL-1β release from human LPS-primed brain slices in a concentration-dependent manner with nanomolar affinity (Fig. 6C and Supplementary Table 4).

**Figure 6:**
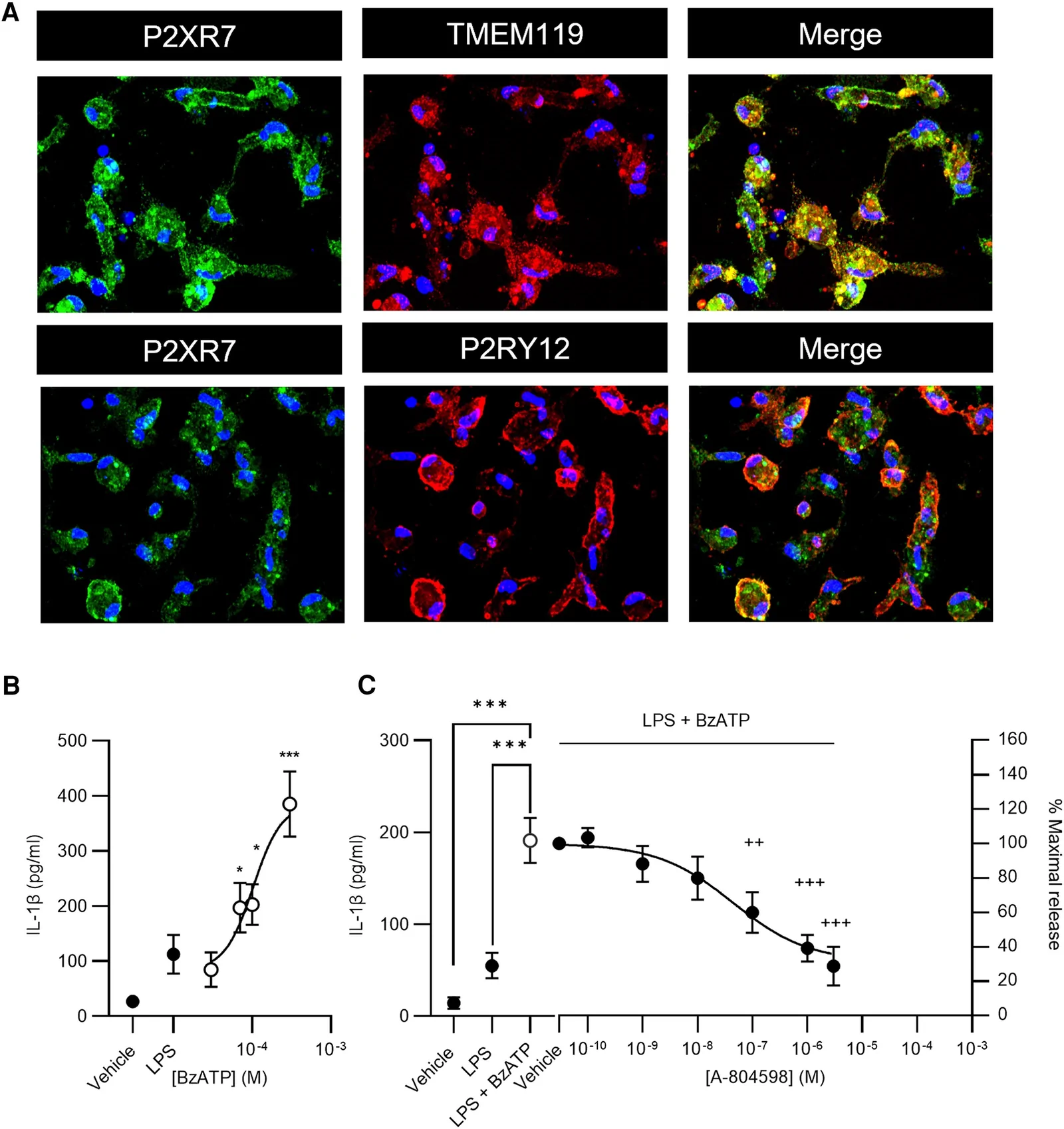
**Concentration-dependent antagonism by the selective P2X7 receptor antagonist A-804598 of BzATP-induced IL-1β release from LPS-primed human brain slices**. Human fresh brain tissue was dissociated and cultured overnight before immunolabelling for TMEM-119, P2X7 receptor and P2RY12 receptor immunoreactivity. (**A**) Representative confocal images displaying indicated immunolabelling of dissociated brain cells with P2X7 receptor (green), TMEM119 (red) and P2RY12 receptor (red). (**B**) Concentration-dependent ability of BzATP to evoke IL-1β release (in picograms per millilitre) from human LPS-primed (100 ng/ml) brain slices [donor tissue was obtained from ≥12 donors; mean ± standard error of the mean (SEM)]. (**C**) Three circles to the left (*left y*-axis) display IL-1β levels in the brain tissue slice culture supernatants (in picograms per millilitre; mean ± SEM), and the percentage maximal release (*right y*-axis; maximum to LPS + BzATP (open circle; 70 µM BzATP) was subjected to iterative curve fitting according to a four-parameter logistic equation. Data are expressed as the mean ± SEM from six independent donors. In **B**, significance was determined using a one-way ANOVA with Dunnett’s *post hoc* test for comparison of picograms per millilitre BzATP-induced IL-1β release compared with vehicle control or in **C**, a one-way ANOVA with Dunnett’s *post hoc* test was carried out for comparison of picograms per millilitre BzATP-induced IL-1β release compared with either LPS or vehicle control (*left y*-axis), or for comparison of the percentage IL-1β release at each concentration of antagonist relative to vehicle control (set to 100%). (**B**) Significant difference of addition of BzATP treatment from vehicle control: ***P < 0.001. (**C**) Significant difference between indicated data: *P < 0.05, ***P < 0.001. Significant difference from LPS + BzATP: ^++^*P <* 0.01 and ^+++^*P <* 0.01. BzATP = 2′(3′)-*O*-(4-benzoylbenzoyl) ATP; IL = interleukin; LPS = lipopolysaccharide.

To investigate the potential of BzATP to impact the release of various soluble factors, a broad panel was used to quantify biomarker release from human brain slices (Fig. 7 for percentage release; for levels expressed as picograms per millilitre see Supplementary Table 5). LPS evoked the release of various biomarkers (e.g. IL-1β, IL-6, TNF-α, MIP-1β and IL-10; Fig. 7A–C), and the additional presence of a near-maximal concentration of BzATP further enhanced release of various biomarkers (e.g. IL-1α, IL-1β, IL-6, GRO-α, LIF, IP-10, SDF-1-α and HGF; Fig. 7A–C), although no apparent impact of BzATP stimulation upon neurofilament light (NfL) release was evident (Supplementary Fig. 4) over the relatively short time course of the stimulation. Furthermore, pretreatment with the P2X7R antagonist A-804598 (3.0 μM) reduced the BzATP-evoked release of IL-1α, IL-1β, GRO-α, IP-10, IL-10 and HGF (Fig. 7A–C).

**Figure 7:**
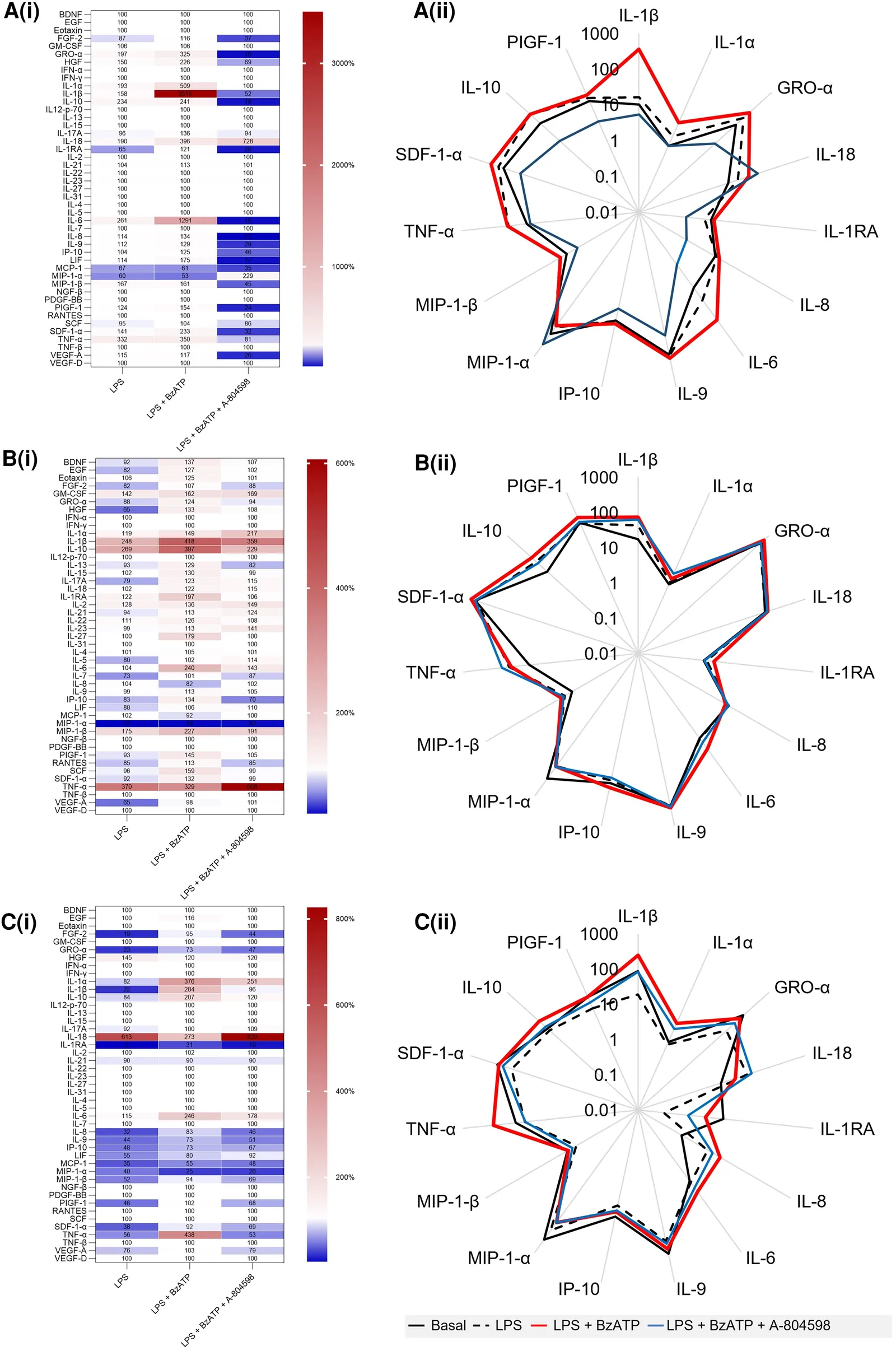
**P2X7 receptor-dependent cytokine release from human brain slices.** Human brain slices were treated in the absence (vehicle) or presence of LPS (100 ng/ml; 2 h) before application of vehicle or BzATP (100 μM) in the absence (vehicle) or presence of the P2X7 receptor antagonist A-804598 (3.0 μM). Heat maps [**A**(**i**), **B**(**i**) and **C**(**i**)] show the percentage change in cytokine levels relative to vehicle control (i.e. no LPS priming), and radar plots [**A**(**ii**), **B**(**ii**) and **C**(**ii**)] show cytokine release in in picograms per millilitre (except for IL-1RA, IL-6, IL-8 and MIP-1- β levels shown as nanograms per millilitre) from cultures treated with vehicle (black solid line; ‘basal’), LPS (black dashed line), LPS + BzATP (red solid line) or LPS + BzATP + A-804598 (blue solid line). Data show three independent donors {Donor 1 [**A**(**i** and **ii**)], Donor 2 [**B**(**i** and **ii**)] and Donor 3 [**C**(**i** and **ii**)]}, expressed as the mean percentage fold change derived from triplicate wells (except for Donor 3, where data are shown originating from duplicate wells). BzATP = 2′(3′)-*O*-(4-benzoylbenzoyl) ATP; IL = interleukin; LPS = lipopolysaccharide; MIP = macrophage inflammatory protein.

Given that the human brain tissue used in these studies was from patients undergoing neurosurgery, we hypothesized that the surgical procedure itself might prime the resected tissue. Indeed, brain tissue slices from the majority of donors were responsive to BzATP in terms of IL-1β release (Fig. 8A), and the BzATP-evoked release was antagonized fully by pretreatment with JNJ-54175446 in a concentration-dependent manner, yielding an apparent affinity in the nanomolar range (Fig. 8B and C and Supplementary Table 3). Overall, these findings are in accordance with those evident in our hMDM model and provide further translational evidence for the importance of the role of the human P2X7R in driving central pro-inflammatory cytokine release, and antagonist action by selective, CNS-penetrant P2X7R antagonists.

**Figure 8:**
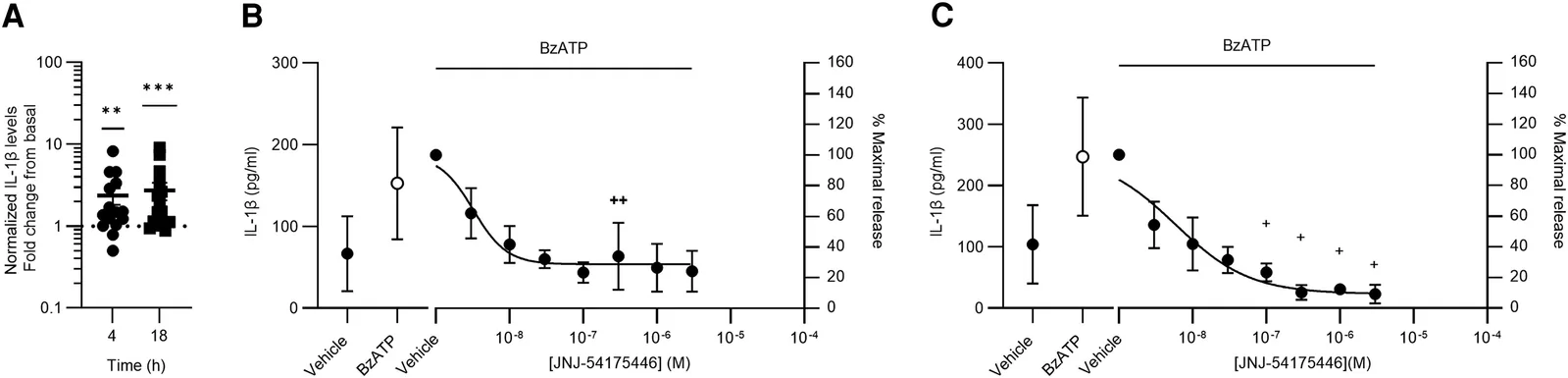
**Concentration-dependent ability of the P2X7 receptor antagonist JNJ-54175446 to antagonize BzATP-induced IL-1β release from human brain slices.** (**A**) IL-1β release from human brain slice cultures treated with BzATP (70 μM); levels display normalized fold change relative to vehicle control (data points represent 15 individual donors), after 4 h (filled circles) or 18 h (filled squares) application of BzATP. (**B** and **C**) Two circles [*left y*-axis; mean ± standard error of the mean (SEM)] display IL-1β levels in the culture supernatants (in picograms per millilitre), and curve fits (*right y*-axis; mean ± SEM) show the impact of JNJ-54175446 upon percentage maximal release relative to BzATP response (open circle; 70 µM BzATP) at 4 h (**B**) and 18 h (**C**). Data in **B** and **C** are expressed as the mean ± SEM from five independent donors. In **A**, a non-parametric Wilcoxon signed-rank test was carried out for comparison of BzATP-induced IL-1β release compared with basal release (set to one) or, in **B** and **C**, a non-parametric Kruskal–Wallis test with Dunn’s *post hoc* test was carried out for comparison of the percentage IL-1β release at each concentration of P2X7 antagonist to vehicle control at 4 h and 18 h (set to 100%). (**A**) Significant increase: **P < 0.01 and ***P < 0.001. (**C**) Significant difference from BzATP: ^+^*P <* 0.05 and ^++^*P <* 0.01. BzATP = 2′(3′)-*O*-(4-benzoylbenzoyl) ATP; IL = interleukin.

## Discussion

The present study provides direct translational evidence from human cellular and brain tissue models to support the clinical use of P2X7R antagonists to limit secondary ATP-driven neuroinflammatory events, such as those that occur in TBI, and thus might improve the clinical outcomes for patients.

Although several trials have investigated the potential clinical benefits of P2X7R antagonists in patients with inflammatory diseases (inflammatory bowel disease^114,115^ or rheumatoid arthritis^84,116,117^), the development of CNS-penetrant P2X7R antagonists^82,86,87,90,118,119^ enables targeting of the P2X7R in neuroinflammatory conditions. We provide data from two independent human translational neuroinflammatory models that support the use of JNJ-54175446 and JNJ-55308942 to inhibit pro-inflammatory cytokine release in neuroinflammation. In an induced human microglial *in vitro* model, we demonstrated inhibition of pro-inflammatory cytokine release by three structurally diverse yet selective P2X7R antagonists (A-804589, JNJ-54175446 and JNJ-55308942). Effective concentrations of these antagonists were in good agreement with their affinities for the P2X7R,^86,87,91,120^ effective concentrations for inhibiting cytokine release^73,86,88,89,91,120^ and, for JNJ-54175446 and JNJ-55308942, achievable brain concentrations from animal pharmacokinetic studies.^121,122^ These data confirm that P2X7R antagonists, including two clinical-stage molecules with ability to penetrate the BBB, display target concentration relevant inhibition of human microglial P2X7R-driven inflammatory cytokine release.

With increasing evidence demonstrating the contribution of microglia in neuroinflammatory pathology, such as in TBI,^18-21,123-125^ and being implicated in neurodevelopmental and neurodegenerative disorders,^55,126^ major importance rests on the generation of human translational models that more closely recapitulate microglial dysfunction in disease whilst also retaining their phenotypic and epigenetic features.^98,99^ In the present study, we have applied an hMDM model adapted from previous reports^103,105,107-112^ to generate translational evidence supporting the use of brain-penetrant P2X7R antagonists in neuroinflammation, which performed as a functionally relevant model predicting drug action in human brain slices. Mechanistically, as expected, the pathway of IL-1β release by hMDM exhibited concentration-dependent inhibition by the selective NLRP3 inhibitor MCC950,^127^ which specifically targets the nucleotide-binding domain of the NLRP3 complex, preventing the hydrolysis of ATP and thus inhibiting NLRP3-induced apoptosis-associated speck-like protein containing a C-terminal caspase recruitment domain (ASC) oligomerization and subsequent cleavage of caspase-1,^127,128^ and also the caspase-1 inhibitor VX-765.^129^ However, the apparent lower level of maximal inhibition by the caspase-1 inhibitor VX-765 might implicate other additional caspases (or pathways) also being involved in IL-1β processing and release.^130-134^ Collectively, these results confirm that hMDM might offer a useful drug screening platform for testing pharmacologically active compounds targeting the P2X7R expressed on human microglia and relevant NLRP3 inflammasome/caspase-mediated cytokine release mechanisms contributing to the neuroinflammatory response evident in the human brain resulting from various pathologies.

Importantly, we extended our findings from the hMDM model to a second *ex vivo* model that we consider a closer representation to the clinical pathology of TBI and other neuroinflammatory diseases. This second optimized methodology uses human precision-cut brain sections from patients undergoing neurosurgical resections. At concentrations in good concordance with the hMDM model, BzATP evoked a pro-inflammatory cytokine release profile from human primed brain sections (Fig. 7) and with largely equivalent potency to promote IL-1β release (Fig. 6B). Confirmation of the role for the P2X7R came from the concentration-related ability of the selective P2X7R antagonist A-804589 to prevent the BzATP-evoked response at pharmacologically relevant concentrations (Fig. 6C).

Previous work using rodent models of neurotrauma,^64,75-81,135^ and the known association of elevated brain cytokine levels, such as IL-1β, in patients with TBI and subsequent detrimental clinical outcomes,^37,136^ emphasizes the relevance of the data presented in this paper, which supports the evaluation of P2X7R antagonists to improve the clinical outcomes of patients following TBI. We believe arguments can be made either way concerning the evaluation of a P2X7R antagonist that crosses the intact BBB. Thus, it might be considered that as the integrity of the BBB is reduced around the injured site after TBI, use of a drug that does not cross the intact BBB would limit brain exposure to areas of injury and thus avoid potential side effects that might arise from exposure to other areas of the brain. Alternatively, it might be considered pertinent to use a P2X7R antagonist that readily crosses the BBB, unlike AZD9056 and CE-224,535.^85^ The P2X7R antagonist JNJ-54175446 was developed to penetrate the BBB and has demonstrated an acceptable safety profile in early-phase clinical studies,^88,89,92^ hence it offers a suitable candidate to evaluate the therapeutic benefit for patients suffering with TBI. This was further evident by the activity of JNJ-54175446 in our hMDM model to inhibit P2X7R-mediated pro-inflammatory cytokine release, which builds on previous work where JNJ-54175446 reduced P2X7R-mediated IL-1β release in a whole blood assay,^88,89^ where the IL-1β is likely to originate from monocytes.^74^ Our studies further extended the evaluation of JNJ-54175446 to demonstrate reversal of BzATP-induced IL-1β release from human brain slices (Fig. 8), again effective concentrations of JNJ-54175446 were in the nanomolar range consistent with the known affinity of JNJ-54175446 for the human P2X7R.^87^ The known impact of IL-1β to promote apoptosis,^137^ further pro-inflammatory cytokine release,^138^ stimulation and recruitment of peripheral leucocytes^139^ and damage of endothelial tight junction proteins leading to BBB breakdown^140^ adds additional support to the hypothesis that P2X7R antagonists^82,86,87,90^ would deliver clinical benefit to patients with TBI. Importantly, we extended our findings with a modification of our human brain slice model to evaluate the potential of the brain-penetrating P2X7R antagonist, JNJ-54175446. The modification in the methodology was the omission of the LPS-priming step, because it was reasoned that the ‘physical insult’ of neurosurgical resection and slicing of the tissue would be sufficient to prime the tissue, to some extent replicating a physical trauma as occurs in TBI.^5^ This appeared to be the case, because BzATP was able to evoke IL-1β release from these brain slices in the majority of samples from donors (Fig. 8A) (note that in the hMDM model BzATP was essentially ineffective to evoke IL-1β release from non-LPS-primed hMDM; Fig. 2). This BzATP-evoked IL-1β release in the human brain slices was antagonized in a concentration-dependent manner at both early (4 h) and more prolonged (18 h) time points (Fig. 8B and C), importantly again, at clinically relevant JNJ-54175446 concentrations that have been predicted to be achieved in early-stage clinical studies in depression.^89^

## Conclusion

Overall, our findings with both human cells differentiated from peripheral blood monocytes that displayed the phenotype and functional characteristics of microglia (hMDM) and also slices of human brain tissue, demonstrated that P2X7R activation (as can occur in the brain following cell damage caused by TBI) promoted a pro-inflammatory response that was prevented by P2X7R antagonists and inhibitors of NLRP3/caspase-1. Such findings support evaluation of relevant pharmacological agents for their ability to improve the clinical outcomes of patients following TBI and other disorders with a neuroinflammatory pathology, such as depression,^125^ schizophrenia,^141,142^ multiple sclerosis,^143^ motor neuron disease,^144^ epilepsy^113^ and, perhaps, neuropathic pain.^145,146^

## Supplementary Material

awag068_Supplementary_Data

## Data Availability

All data that support the findings of this study are present within the article. Data were generated at the University of Birmingham and Celentyx Ltd and are available from the corresponding author upon reasonable request. All materials are commercially available, except for JNJ-54175446.
