# Supplementary material for: P2X7 receptor-mediated IL-1β release by human brain tissue: the impact of CNS-penetrant potential therapeutics
Source: Brain. 2026 Feb 19;149(10):3614–30. doi: 10.1093/brain/awag068 (PMC13634649; doi:10.1093/brain/awag068)
Supplement: Supplementary file 1 [file awag068_supplementary_data.pdf]

Supplementary Material 1

Methods for supplementary data

hMDM labelling by flow cytometry

hMDM differentiated for five days were blocked with PBS containing 5% human AB serum (Complement technologies) and were surface labelled with a direct conjugate anti-HLA-DR antibody (Biolegend; 1:200) for 0.5 h at RT. Following two washes, hMDM were fixed with cytofix fixation buffer (BD Biosciences) and were permeabilised (eBioscience), followed by intracellular labelling with direct conjugate antibodies against CX3CR1 (Biolegend; 1:200), Iba1 (Biolegend; 1:200) and P2RY12 (Biolegend; 1:200) for 0.5 h at RT. Cells were washed and were acquired using a Beckmann coulter Cytoflex flow cytometer.

hMDM phagocytosis assays

Labelling myelin basic protein

Human myelin basic protein (MBP; Enzo) was labelled with pHrodo-red SE dye (molar ratio of 1.0 mole of protein to 1.0 mole of dye; Thermofisher), for 45 minutes at room temperature in the dark. Excess unreacted dye was removed using Zeba dye removal spin columns (Thermofisher).

Phagocytosis assay

After 5-7 days, hMDM were pre-treated in presence of relevant vehicle (DMSO; 0.06%) or presence of cytochalasin-D (10 μM; Sigma) for 2 hours. During the last 30 minutes of incubation, hMDM were labelled with calcein green dye (10 nM; Thermofisher). Following pre-incubation, hMDM were treated with pHrodo-red labelled MBP (100 μg/mL), and were imaged using high-content confocal imaging (Yokogawa CQ1 imaging platform; x 40 objective lens, imaging at least five fields of view per well), every 20 minutes for a total period of 6 hours. MBP uptake within calcein positive cells was quantified as total area per cell, using CQ1 image analysis software (version 1.02.02.00).

Immunocytochemistry

After 5-7 days of culture, hMDM cells were primed with LPS (100 ng/mL; InvivoGen) for 2.5 hours, prior to 4 hours treatment with BzATP (Sigma) or vehicle (water; 0.14%). hMDM were fixed with 4% paraformaldehyde for 12 minutes and washed twice with PBS. Cells were

permeabilized with 0.1% Triton-X-100 (Sigma) for 10 minutes, blocked with 5% goat serum (Sigma) in PBS + 0.1% Tween-20 (Sigma) for 1 hour and labelled with P2X7R (DSHB; 1:100) and Iba1 (Invitrogen; 1:300) primary antibodies at room temperature for 1 hour. After washing with PBS (Sigma), cells were incubated with secondary antibodies at room temperature in the dark for 1 hour. Cells were imaged and immunofluorescence quantified using high-content confocal imaging (Yokogawa CQ1 imaging platform; x 40 objective lens, imaging at least nine fields of view per well). Positive cell surface immunolabelling on cells was quantified as total intensity per cell, using CQ1 image analysis software (version 1.02.02.00).

Neurofilament light chain ELISA

Supernatants were harvested from human brain slice cultures stimulated as described in the human brain slice culture experiments section of the main manuscript materials and methods. Supernatants were centrifuged at 1,000 x g for 5 minutes to pellet debris. NfL levels in the cell-free supernatants were determined using a human Neurofilament light chain (NfL) ELISA kit (Abbexa), as per manufacturers instructions.

Supplementary Table 1. Demographics, clinical and pathological data of human brain tissue donors used in this study. All tissue samples except for P2X70013 and P2X70014 were sampled beyond the tumoral bed, within the margin which appeared radiologically normal by MRI.

Sample code	Age range	Sex	Primary pathology	Grade	Tissue type sampled
P2X70004	60-75	M	Primary glioma	Grade 4	Cortical access
P2X70006	60-75	F	Primary glioma	Grade 4	Cortical access
P2X70007	60-75	M	Cerebellar metastasis	Metastasis	Cortical access (cerebellum)
P2X70010	40-59	M	Primary glioma	Grade 3	Cortical access
P2X70013	40-59	F	Primary glioma	Grade 3	Tumour bed margin
P2X70014	18-39	M	Primary glioma	Grade 3	Tumour bed margin
P2X70015	60-75	M	Primary glioma	Grade 4	Cortical access
P2X70023	60-75	M	Cerebellar metastasis	Metastasis	Cortical access (cerebellum)
P2X70026	40-59	M	Primary glioma	Grade 3	Cortical access
P2X70030	40-59	F	Primary glioma	Grade 3	Cortical access
P2X70034	60-75	F	Chiari malformation	Benign	Cerebellar tonsil
P2X70041	40-59	F	Cerebral metastasis	Metastasis	Cortical access
P2X70045	40-59	F	Cerebral metastasis	Metastasis	Cortical access
P2X70046	18-39	F	Chiari malformation	Benign	Cerebellar tonsil
P2X70048	18-39	F	Chiari malformation	Benign	Cerebellar tonsil
P2X70050	40-59	M	Primary glioma	Grade 4	Cortical access
P2X70051	40-59	M	Cerebral metastasis	Metastasis	Cortical access
P2X7055	60-75	M	Primary glioma	Grade 4	Cortical access
P2X70056	40-59	M	Primary glioma	Grade 4	Cortical access
P2X70057	60-75	F	Chiari malformation	Benign	Cerebellar tonsil
P2X70058	18-39	F	Chiari malformation	Benign	Cerebellar tonsil
P2X70065	60-75	F	Primary glioma	Grade 4	Cortical access
P2X70068	40-59	F	Primary glioma	Grade 4	Cortical access
P2X70075	18-39	M	Primary glioma	Grade 4	Cortical access
P2X70076	18-39	F	Chiari malformation	Benign	Cerebellar tonsil
P2X70077	40-59	M	Primary glioma	Grade 2	Cortical access
P2X7078	18-39	M	Primary glioma	Grade 4	Cortical access

Supplementary Table 2. Pharmacological values for the P2X7 receptor agonist, BzATP, P2X7 receptor antagonists, A-804598, JNJ-54175446 and JNJ-55308942, and inhibitors, MCC950 (NLRP3 inflammasome inhibitor) and VX-765 (caspase-1 inhibitor), generated using the hMDM model. Data (mean±SEM) from at least five independent experiments.

Drug	**IL-1β release pEC_{50}**	**IL-1β release pIC_{50}**	**IL-18 release pEC_{50}**	**IL-18 release pIC_{50}**
**BzATP**	4.04±0.05		3.95±0.02	
**A-804598**		8.09±0.25		7.83±0.36
**JNJ-54175446**		8.92±0.14		8.73±0.28
**JNJ-55308942**		8.18±0.08		8.22±0.10
**MCC-950 Sodium**		8.62±0.07		8.61±0.06
**VX-765**		5.46±0.58		5.03±0.48

Supplementary Table 3. Levels of soluble biomarker release from hMDM. hMDM cultures were primed with LPS (100 ng/mL) with subsequent addition of the non-selective P2X7 receptor agonist, BzATP (100 μM) in the absence (vehicle) or presence of the selective P2X7 receptor antagonist, A-804598 (3.0 μM). Analyte concentration for donors 1-3 is depicted as mean picogram per millilitre levels. Data is derived from triplicate culture wells.

	**Donor 1**				**Donor 2**				**Donor 3**			
	**Control**	**LPS**	**LPS + BzATP**	**LPS + BzATP + A-804598**	**Control**	**LPS**	**LPS + BzATP**	**LPS + BzATP + A-804598**	**Control**	**LPS**	**LPS + BzATP**	**LPS + BzATP + A-804598**
**BDNF**	4.6	6.1	5.1	4.6	5.1	5.9	5.3	5.3	6.1	7.6	6.4	6.2
**EGF**	35.2	70.1	63.8	58.5	37.6	48.4	41.6	42.1	63.6	86.2	76.0	105.3
**Eotaxin**	2.4	3.3	3.0	2.7	4.3	5.1	4.6	4.4	4.9	6.1	5.0	5.9
**FGF-2**	6.4	28.4	13.4	6.4	6.4	30.7	6.4	6.4	6.4	35.2	6.4	6.4
**GM-CSF**	6954.5	5332.7	7387.7	7846.5	6910.0	4597.4	7687.6	4479.7	2398.9	2825.9	3157.9	5181.1
**GRO-a**	328.9	694.5	459.1	445.8	230.0	501.0	325.3	239.9	341.1	973.8	599.8	793.7
**HGF**	171.2	188.4	212.5	188.4	82.7	85.0	91.9	87.9	80.0	102.3	102.2	115.3
**IFN-a**	1.6	1.6	1.6	1.6	1.6	1.6	1.6	1.6	1.6	1.6	1.6	1.6
**IFN-g**	18.9	18.9	18.9	18.9	18.9	18.9	18.9	18.9	18.9	18.9	18.9	18.9
**IL-1a**	1.1	4.9	61.3	3.9	1.1	2.5	20.6	1.5	1.1	5.7	44.7	4.3
**IL-1b**	25.0	261.7	4527.2	242.2	17.0	121.4	2622.9	113.7	16.1	195.0	4168.0	227.1
**IL-10**	3.1	151.2	16.2	24.5	3.1	29.5	6.9	8.8	3.1	27.5	9.8	13.3
**IL12-p-70**	15.9	15.9	15.9	15.9	15.9	15.9	15.9	15.9	15.9	15.9	15.9	15.9
**IL-13**	8.3	8.3	8.3	8.3	23.3	24.9	24.3	17.0	23.9	27.5	31.0	31.5
**IL-15**	5.9	5.9	5.9	5.9	11.0	18.8	17.3	14.7	17.5	25.9	20.1	19.1
**IL-17A**	31.3	35.7	36.8	33.1	27.2	27.7	30.8	26.6	27.5	34.7	31.7	29.7
**IL-18**	36.1	64.0	245.7	61.3	41.6	59.3	222.1	55.6	53.6	92.3	242.8	83.6
**IL-1RA**	214955.9	212862.8	289583.4	252071.5	176831.0	160365.0	191999.0	171555.9	285951.2	242058.7	196102.8	238491.4
**IL-2**	19.7	79.7	40.4	42.6	19.7	48.5	22.9	21.4	19.7	76.7	49.2	56.0
**IL-21**	36.5	77.5	67.3	60.9	36.9	60.4	49.0	47.7	52.2	95.2	73.6	83.4
**IL-22**	48.4	61.2	56.1	52.3	48.4	51.3	49.6	54.2	48.4	71.9	56.0	54.0
**IL-23**	30.0	150.7	81.1	117.6	30.0	73.7	51.2	35.8	30.0	314.2	180.5	355.4
**IL-27**	38.3	38.3	38.3	38.3	38.3	78.8	38.3	38.3	38.3	78.8	38.3	38.3
**IL-31**	24.3	24.3	24.3	24.3	24.3	24.3	24.3	24.3	24.3	24.3	24.3	24.3
**IL-4**	55.6	61.0	70.2	64.0	65.1	62.3	70.7	51.1	45.5	65.8	62.7	81.1
**IL-5**	20.1	28.7	20.1	20.1	20.1	22.5	20.1	20.1	20.1	40.9	20.1	20.1
**IL-6**	50.8	59124.1	13823.2	25969.0	69.4	13600.0	6083.5	8180.4	1748.6	105000.0	105000.0	105000.0
**IL-7**	1.4	1.4	1.4	1.4	1.4	1.4	1.4	1.4	1.4	2.0	1.4	1.4
**IL-8**	6966.2	10007.6	6807.3	6912.9	9336.2	9247.6	10306.0	7481.8	18588.8	13270.3	9258.2	10439.2
**IL-9**	298.2	331.0	322.0	284.4	305.2	328.3	326.9	285.7	334.5	306.9	323.9	373.1
**IP-10**	25.6	38.9	33.6	32.4	23.1	27.0	25.0	23.8	25.2	32.3	30.6	32.0
**LIF**	20.6	27.1	28.3	27.4	20.1	24.3	24.2	23.0	20.6	39.5	36.7	46.5
**MCP-1**	1037.1	1168.8	1132.7	1045.3	1198.1	1344.5	1443.0	1258.4	1575.8	1424.6	1575.6	1356.2
**MIP-1a**	477.6	106.3	94.3	97.0	273.2	112.9	113.0	110.0	150.2	100.9	105.9	95.0
**MIP-1b**	1715.7	64000.0	7509.2	28695.9	1281.7	4566.1	2842.6	2764.9	2956.2	64000.0	64000.0	64000.0
**NGF-b**	13.0	13.0	13.0	13.0	13.0	13.0	13.0	13.0	13.0	13.0	13.0	13.0
**PDGF-BB**	9.0	9.0	9.0	9.0	9.0	9.0	9.0	9.0	9.0	9.0	9.0	9.0
**PIGF-1**	86.6	171.4	127.9	121.6	57.5	106.9	80.6	62.2	75.0	203.0	143.4	181.8
**RANTES**	3.2	11.4	8.2	9.1	4.3	7.7	6.1	5.3	5.3	10.9	8.9	11.4
**SCF**	3.0	3.4	3.8	3.2	3.3	4.1	3.6	3.4	3.1	4.3	3.5	3.8
**SDF-1a**	452.3	843.7	638.8	672.8	335.9	708.8	473.8	375.7	457.4	1149.8	840.9	987.6
**TNF-a**	58.5	2624.8	1138.2	1203.1	47.0	1108.3	202.6	221.8	43.7	3054.7	1143.0	1973.2
**TNF-b**	12.8	19.0	15.6	12.8	12.8	12.8	12.8	12.8	12.8	12.8	12.8	12.8
**VEGF-A**	108.9	144.3	123.2	129.8	84.7	117.9	102.1	98.9	118.6	176.9	140.1	164.4
**VEGF-D**	3.7	3.9	3.7	3.8	3.7	3.7	3.7	3.7	3.7	3.7	3.7	3.7

Supplementary Table 4. Pharmacological values for the P2X7 receptor agonist, BzATP, P2X7 receptor antagonists, A-804598 and JNJ-54175446, generated using slices of human brain. Data (mean±SEM) from at least five independent experiments.

Drug	**IL-1β release pEC_{50} (4 h)**	**IL-1β release pIC_{50} (4 h)**	**IL-1β release pIC_{50} (18 h)**
**BzATP**	3.69±0.30		
**A-804598**		7.46±0.25	
**JNJ-54175446**		8.30±0.15	8.22±0.20

Supplementary Table 5. Levels of soluble biomarker release from human brain slice cultures. Human brain slice cultures were primed with LPS (100 ng/mL) with subsequent addition of the non-selective P2X7 receptor agonist, BzATP (100 µM) in the absence (vehicle) or presence of the selective P2X7 receptor antagonist, A-804598 (3.0 µM). Analyte concentration for donors 1-3 is depicted as mean picogram per millilitre levels. Data is derived from at least three culture wells.

	**Donor 1**				**Donor 2**				**Donor 3**			
	**Control**	**LPS**	**LPS + BzATP**	**LPS + BzATP + A-804598**	**Control**	**LPS**	**LPS + BzATP**	**LPS + BzATP + A-804598**	**Control**	**LPS**	**LPS + BzATP**	**LPS + BzATP + A-804598**
**BDNF**	4.1	4.1	4.1	4.3	5.1	4.7	7.0	5.4	4.1	4.1	4.1	4.1
**EGF**	5.4	5.4	5.4	7.8	14.7	12.0	18.7	15.0	5.4	5.4	6.2	5.4
**Eotaxin**	1.3	1.3	1.3	2.1	3.8	4.1	4.8	3.9	1.3	1.3	1.3	1.3
**FGF-2**	17.2	14.9	20.0	81.7	229.3	187.5	245.6	201.8	129.9	24.2	123.0	56.8
**GM-CSF**	41.3	43.7	43.8	43.7	56.8	80.5	92.2	95.9	41.3	41.3	41.2	41.3
**GRO-a**	44.6	87.9	145.0	236.1	527.6	462.5	656.7	498.0	105.4	24.0	77.4	49.3
**HGF**	18.0	27.0	40.8	39.2	61.0	39.6	81.4	65.7	12.5	18.1	15.0	15.0
**IFN-a**	1.6	1.6	1.6	1.6	1.6	1.6	1.6	1.6	1.6	1.6	1.6	1.6
**IFN-g**	18.9	18.9	18.9	18.9	18.9	18.9	18.9	18.9	18.9	18.9	18.9	18.9
**IL-1a**	1.1	2.1	5.5	2.1	1.4	1.7	2.1	3.1	1.3	1.1	5.0	3.3
**IL-1b**	10.3	16.3	363.2	73.4	17.6	43.7	73.8	63.3	88.9	19.2	252.8	85.0
**IL-10**	52.0	121.5	125.5	81.4	29.6	79.4	117.4	67.6	28.6	24.0	59.3	34.4
**IL12-p-70**	15.9	15.9	15.9	15.9	15.9	15.9	15.9	15.9	15.9	15.9	15.9	15.9
**IL-13**	8.3	8.3	8.3	9.7	13.9	12.9	17.9	11.4	8.3	8.3	8.3	8.3
**IL-15**	5.9	5.9	5.9	5.6	6.7	6.8	8.7	6.6	5.9	5.9	5.9	5.9
**IL-17A**	6.6	6.4	9.0	12.5	24.0	18.9	29.4	27.7	6.2	5.7	6.2	6.8
**IL-18**	4.3	8.1	17.0	30.8	62.4	63.9	75.8	72.0	3.0	18.1	8.1	24.5
**IL-1RA**	1079.0	702.6	1301.9	807.0	752.4	918.0	1484.2	796.1	2702.5	55.5	833.2	269.5
**IL-2**	19.7	19.7	19.7	19.7	19.7	25.2	26.9	29.4	19.7	19.7	20.1	19.7
**IL-21**	27.9	28.9	31.4	32.9	43.8	41.3	49.7	54.2	31.5	28.2	28.2	28.2
**IL-22**	48.4	48.4	48.4	48.4	48.4	53.9	61.1	52.4	48.4	48.4	48.4	48.4
**IL-23**	29.9	30.0	30.0	30.0	30.4	30.1	34.4	42.8	30.0	30.0	30.0	30.0
**IL-27**	38.3	38.3	38.3	38.3	38.3	38.3	68.6	38.3	38.3	38.3	38.3	38.3
**IL-31**	24.3	24.3	24.3	24.3	24.3	24.3	24.3	24.3	24.3	24.3	24.3	24.3
**IL-4**	30.3	30.3	30.3	30.3	30.3	30.5	31.7	30.5	30.3	30.3	30.3	30.3
**IL-5**	20.1	20.1	20.1	22.1	28.7	23.0	29.3	32.7	20.1	20.1	20.1	20.1
**IL-6**	4083.9	10672.5	52739.4	10857.7	9344.2	9676.9	22440.7	13399.8	3047.1	3489.3	7506.7	5418.3
**IL-7**	1.4	1.4	1.4	1.6	2.1	1.6	2.2	1.9	1.4	1.4	1.4	1.4
**IL-8**	2912.1	3319.1	3900.8	3251.0	8865.5	9262.1	7293.3	9019.8	5976.7	1926.3	4938.4	2774.6
**IL-9**	117.0	131.1	150.7	156.1	265.0	263.5	299.0	278.5	146.8	63.9	107.5	75.6
**IP-10**	12.4	12.8	15.6	25.7	57.6	47.9	77.4	40.3	12.3	5.9	8.9	8.2
**LIF**	100.5	114.5	175.6	279.1	753.5	663.9	802.2	826.3	33.1	18.2	26.6	30.6
**MCP-1**	2148.0	1435.6	1310.9	1000.2	1603.8	1637.6	1473.9	1604.1	1740.5	605.2	955.5	835.0
**MIP-1a**	160.6	96.2	84.7	166.6	241.1	89.6	92.2	96.0	348.5	165.9	87.5	89.5
**MIP-1b**	2151.6	3597.2	3463.1	2561.9	1444.1	2526.9	3275.2	2752.0	2054.4	1059.1	1927.5	1424.9
**NGF-b**	13.0	13.0	13.0	13.0	13.0	13.0	13.0	13.0	13.0	13.0	13.0	13.0
**PDGF-BB**	9.0	9.0	9.0	9.0	9.0	9.0	9.0	9.0	9.0	9.0	9.0	9.0
**PIGF-1**	25.0	30.9	38.5	53.4	116.5	108.5	169.0	122.2	32.1	14.6	32.8	21.8
**RANTES**	1.7	1.7	1.7	2.4	4.0	3.4	4.5	3.4	1.7	1.7	1.7	1.7
**SCF**	2.3	2.2	2.4	2.8	3.4	3.3	5.4	3.4	2.0	2.0	2.0	2.0
**SDF-1a**	95.5	134.6	222.2	335.1	731.2	669.5	964.2	720.8	158.0	59.5	145.6	108.4
**TNF-a**	14.0	46.5	48.9	20.7	12.7	47.1	41.9	77.4	30.7	17.1	134.3	16.4
**TNF-b**	12.8	12.8	12.8	12.8	12.8	12.8	12.8	12.8	12.8	12.8	12.8	12.8
**VEGF-A**	744.4	854.7	869.0	1321.5	2564.6	1655.2	2516.6	2577.8	12.8	9.8	13.1	10.1
**VEGF-D**	3.7	3.7	3.7	3.7	3.7	3.7	3.7	3.7	3.7	3.7	3.7	3.7

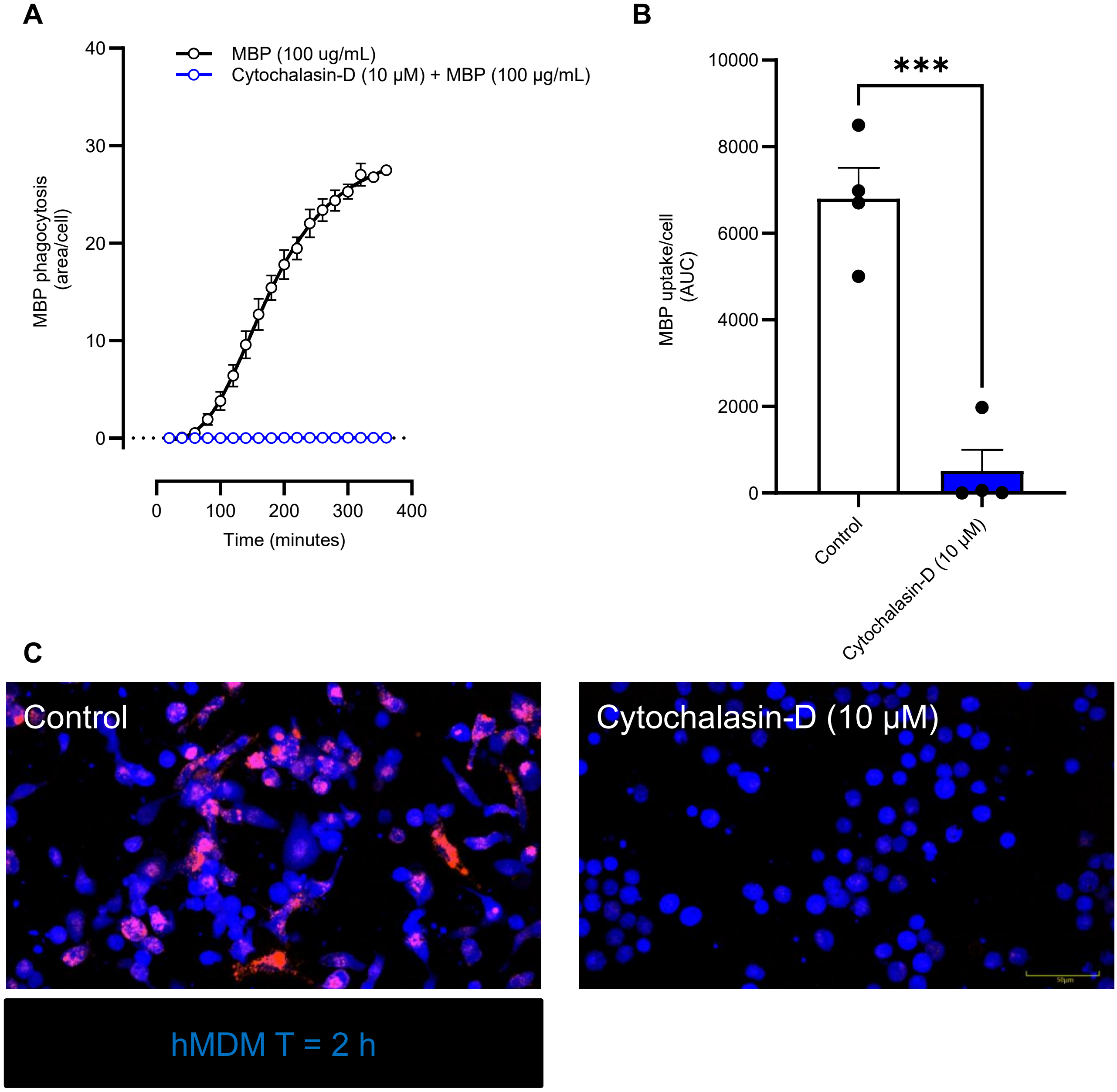
A
MBP (100 ug/mL)
Cytochalasin-D (10 μM) + MBP (100 μg/mL)
MBP phagocytosis (area/cell)
0
10
20
30
40
0
100
200
300
400
Time (minutes)
B
MBP uptake/cell (AUC)
0
2000
4000
6000
8000
10000
***
Control
Cytochalasin-D (10 μM)
C
Control
Cytochalasin-D (10 μM)
hMDM T = 2 h

Supplementary Figure 1. Uptake of myelin basic protein by hMDM. (**A**) Time-course graph showing uptake of myelin basic protein (MBP; 100 µg/mL) over time by hMDM pre-treated for 2 h in the absence (open circles) or presence of cytochalasin-D (10 µM; blue open circles). (**B**) MBP uptake quantified by area under the curve (AUC). (**C**) Representative confocal images of control or cytochalasin-D treated hMDM labelled with calcein (blue) showing uptake of MBP (red) within cells at 2 h. Scale bar: 50 µm. (**A**) Data expressed as mean ± SD derived from a single representative donor and (B) mean + SEM, derived from four independent experiments with cells originating from four different donors. Significance was determined using an unpaired T test. Significant difference between indicated data ***$P<0.001$.

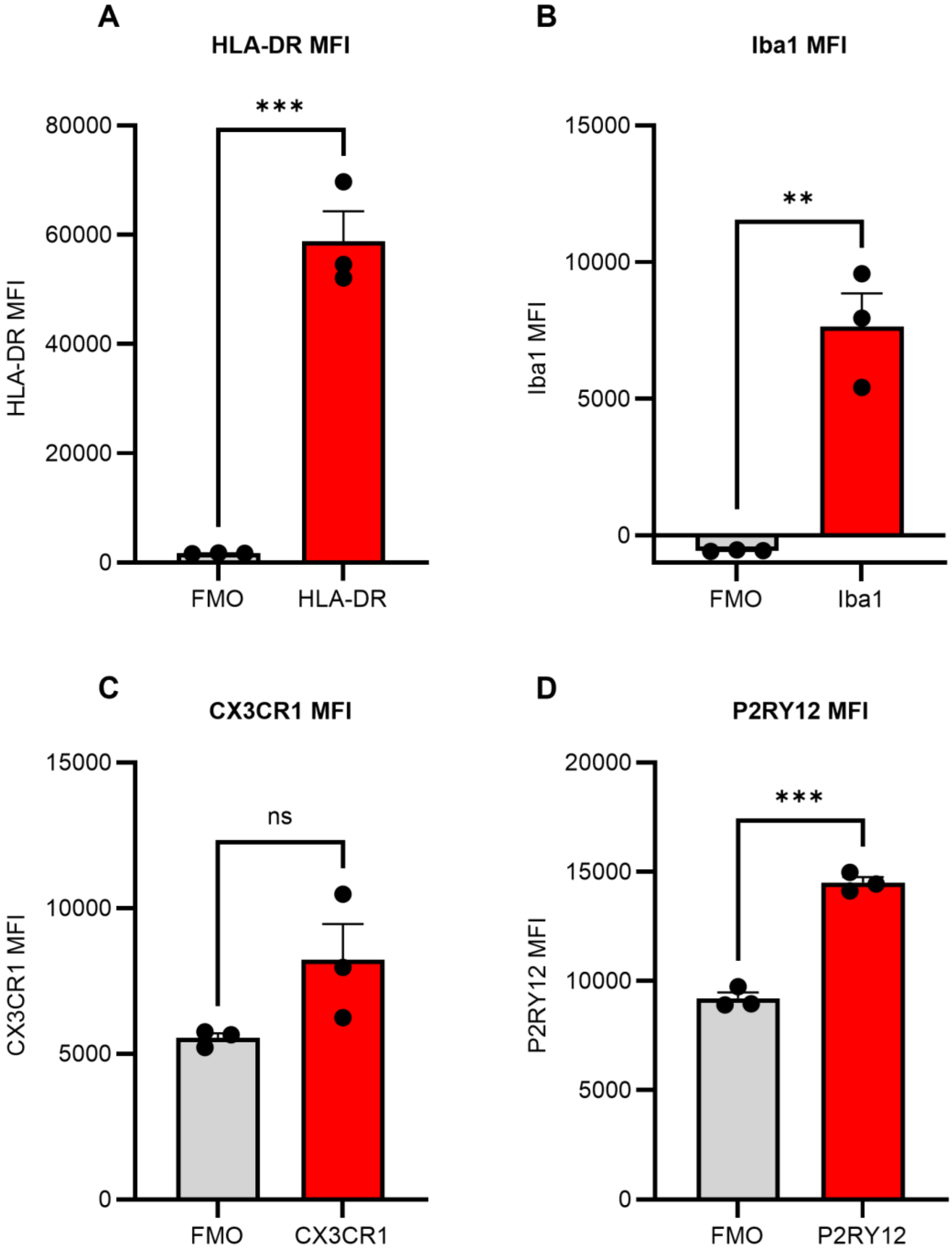

Supplementary Figure 2. Expression of HLA-DR, Iba1, CX3CR1 and P2RY12 on hMDM. hMDM differentiated for five days were stained using antibodies against HLA-DR, Iba1, CX3CR1 and P2RY12 and were acquired by flow cytometry. Expression of HLA-DR (**A**), Iba1 (**B**), CX3CR1 (**C**) and P2RY12 (**D**) by geometric median fluorescent intensity (MFI). FMO = fluorescence minus one control. Data expressed as mean + SEM, and individual points represent three independent experiments with cells originating from three different donors. Significance was determined using an unpaired t test. Significant difference between indicated data $^{**}P<0.01$, $^{***}P<0.001$.

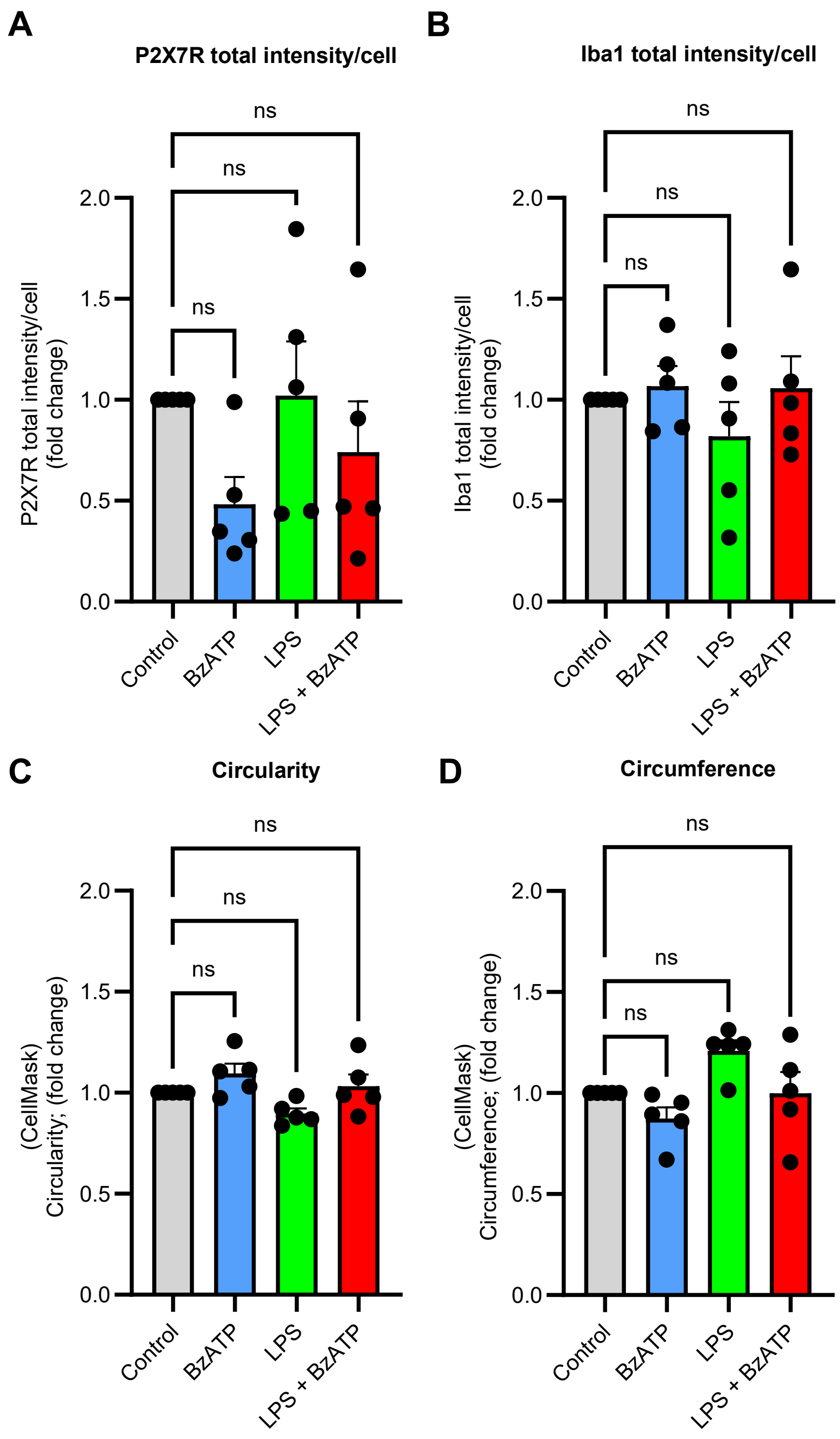

Supplementary Figure 3. Impact of P2X7R stimulation on P2X7R/Iba-1 expression and morphological changes in hMDM. (**A-D**) hMDM cultures were treated in the absence (vehicle) or presence of LPS (100 ng/mL; 2 h) before application of vehicle or BzATP (100 µM; 4 h). Cells were fixed and following permeabilization, were labelled using antibodies against P2X7R and Iba-1 and were imaged using high-content confocal microscopy. (**A-D**) represents quantification of immunostaining total intensity (fold change relative to control cells) for P2X7R (**A**), Iba-1 (**B**), and changes in cell roundness; circularity (**C**) and cell size; circumference (**D**). Cellular morphology analysis utilised the IBA-1 positive cell mask. Data expressed as mean + SEM, and individual points represent five independent experiments with

cells originating from five different donors. Significance was determined using a One-way ANOVA with Tukey's *post hoc* test for comparison of BzATP, LPS, and LPS + BzATP to vehicle control.

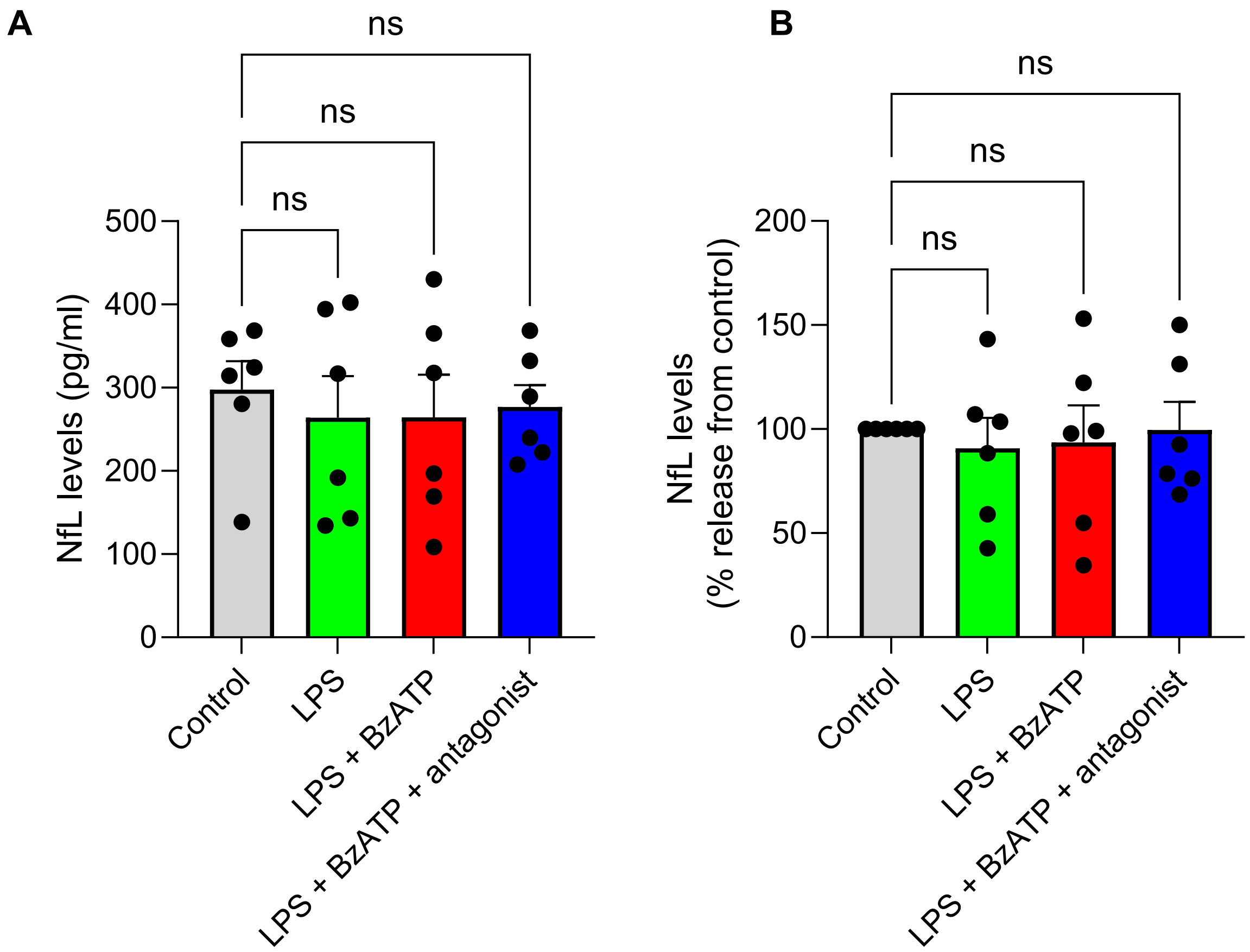

Supplementary Figure 4. NfL release from human brain slice cultures. Human brain slices were treated in the absence (vehicle) or presence of LPS (100 ng/mL; 2 h) before application of vehicle or BzATP (100 µM) in the absence (vehicle) or presence of the P2X7 receptor antagonist (represents JNJ-54175446 and A-804598 treated cultures), for 4 h. NfL levels were monitored by ELISA. (**A-B**) represents mean picograms per millilitre NfL levels (**A**) or mean percentage NfL levels relative to vehicle control treated slices (**B**). Bars are expressed as mean + SEM and individual points represent data obtained from six independent experiments utilising tissue obtained from six different donors. Significance was determined using a One-way ANOVA with Tukey's *post hoc* test for comparison of LPS, LPS + BzATP and LPS + BzATP + P2X7R antagonist to vehicle control treated slices.
